# Demonstrating the source of inherent instability in NiFe LDH-based OER electrocatalysts†

**DOI:** 10.1039/d2ta07261k

**Published:** 2023-01-17

**Authors:** Daire Tyndall, Michael John Craig, Lee Gannon, Cormac McGuinness, Niall McEvoy, Ahin Roy, Max García-Melchor, Michelle P. Browne, Valeria Nicolosi

**Affiliations:** a School of Chemistry, Trinity College Dublin, College Green Dublin 2 Ireland nicolov@tcd.ie; b CRANN and AMBER Research Centres, Trinity College Dublin, College Green Dublin 2 Ireland; c School of Physics, Trinity College Dublin, College Green Dublin 2 Ireland; d Materials Science Centre, Indian Institute of Technology Kharagpur West Bengal 721302 India; e Helmholtz-Zentrum Berlin für Materialien und Energie Berlin 14109 Germany; f I-Form Research, Trinity College Dublin Ireland

## Abstract

Nickel-iron layered double hydroxides are known to be one of the most highly active catalysts for the oxygen evolution reaction in alkaline conditions. The high electrocatalytic activity of the material however cannot be sustained within the active voltage window on timescales consistent with commercial requirements. The goal of this work is to identify and prove the source of inherent catalyst instability by tracking changes in the material during OER activity. By combining *in situ* and *ex situ* Raman analyses we elucidate long-term effects on the catalyst performance from a changing crystallographic phase. In particular, we attribute electrochemically stimulated compositional degradation at active sites as the principal cause of the sharp loss of activity from NiFe LDHs shortly after the alkaline cell is turned on. EDX, XPS, and EELS analyses performed after OER also reveal noticeable leaching of Fe metals compared to Ni, principally from highly active edge sites. In addition, post-cycle analysis identified a ferrihydrite by-product formed from the leached Fe. Density functional theory calculations shed light on the thermodynamic driving force for the leaching of Fe metals and propose a dissolution pathway which involves [FeO_4_]^2−^ removal at relevant OER potentials.

## Abstract 2

During their lifetime, the electrocatalytically active NiFe LDH platelets show impressive, but unstable water-splitting capabilities due largely to compositional degradation.

## Graphical abstract

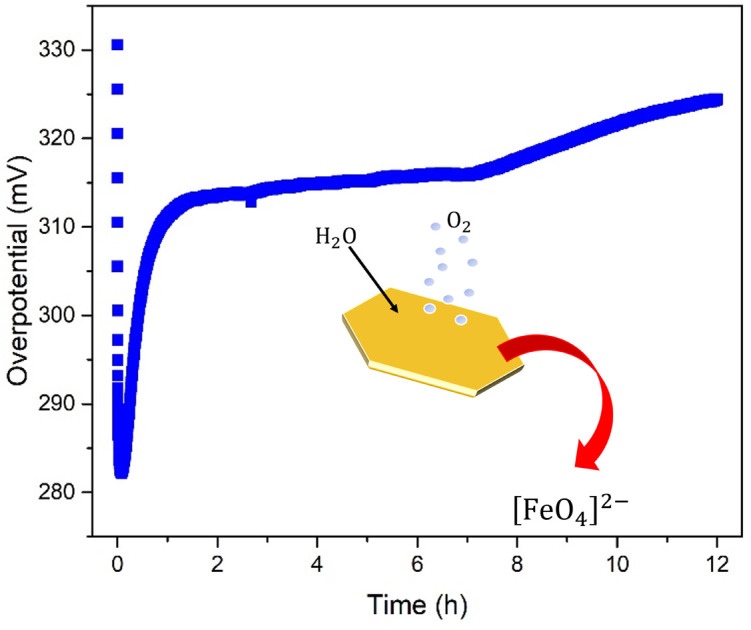

## Introduction

In order to meet the ever-growing global energy demand, alternative routes toward sustainable energy production have been proposed. One such route is the use of green hydrogen gas as a fuel which combusts in air with a high energy density and no harmful by-products. Water-splitting *via* electrolysis can be used to produce green H_2_, a reaction which can be catalyzed in an electrolyzer cell at the cathode (hydrogen evolution) and the anode (oxygen evolution).^2–4^ The hydrogen evolution reaction (HER) is most effectively catalyzed by Pt with close to zero overpotential,^5^ while in the case of the more kinetically sluggish oxygen evolution reaction (OER), such efficiency remains elusive.

Several materials from the layered double hydroxide (LDH) family have been shown to be highly active electrocatalysts for the OER.^6,7^ It is well known, and has been comprehensively reported, that nickel-iron (NiFe) LDH has particularly impressive electrocatalytic capabilities for the OER half-reaction in alkaline media, established by a delicate push–pull mechanism between the Ni and Fe metal centers to favorably distribute electron density for the approach of OH^−^ ions which kick-starts the reaction.^8,9^ NiFe LDHs exhibit low overpotentials (*η*) and Tafel slope values compared with other candidates from the LDH family as well as other competitive metal oxides being researched for this purpose, due largely to the density of highly active sites at platelet edges.^3,10–12^ For example, in a previous work from our group on NiFe LDHs, we reported that these materials under post-synthesis modification could reach OER overpotential values as low as 245 ± 7 mV and a 29 mV dec^−1^ Tafel slope.^13^ Additionally, the material brings with it cost-effectiveness when compared to the state-of-the-art catalysts reported in the literature for alkaline OER, namely RANEY^®^ nickel and precious ruthenium/iridium compounds.^12,14,15^ However, for materials such as LDHs (or others like it) to become high performance catalysts in line with the practical requirements associated with commercial applications, there are a number of considerations aside from the standard figures of merit (*e.g.* overpotential, Tafel slope) which must be faced. One extremely important factor is the stability of the catalyst under highly oxidative conditions, which we address in this work.

Herein, the electrochemical stability of NiFe LDH is characterized in alkaline media at constant current *via* chronopotentiometry (CP) and in a cyclic manner *via* cyclic voltammetry (CV). While some previous studies have shown relatively little decay of the catalyst performance,^16–18^ others have demonstrated a clear loss of activity over time for the pure material.^19–21^ Hence, the aim of this work is to highlight the inherent instability of NiFe LDH catalysts as active components in alkaline water electrolyzer cells and identify the principle degradation mechanisms. This knowledge is essential for the development of future strategies to rectify the shortcomings and realize the full potential of NiFe LDH catalysts for commercial water electrolyzers. To this aim, two fundamental structural parameters, namely crystallographic phase and chemical composition have been investigated as a function of time to observe any change in the NiFe LDH material. To begin with a note on the phase, a scheme has been effectively established for NiFe LDH OER catalysts through a combination of *in situ* experiments as well as comparisons with the phase behaviour of similar hydroxide catalysts.^8,22,23^ Specifically, a direct analogy can be made between the crystallographic phases of NiFe LDH and the ‘Bode scheme’ for Ni(OH)_2_ catalysts (ESI Fig. S1†).^24^ The Bode scheme describes four unique crystal phases (limiting cases), interchangeable by ageing and charging/discharging in active media. There are two hydrated phases, α-Ni(OH)_2_/γ-NiOOH, and two dehydrated phases, β-Ni(OH)_2_/β-NiOOH. It has been demonstrated by Trotochaud *et al.*^25^ that the partial incorporation of Fe^3+^ as metal centres in the hydroxide structure limits the phase scheme to only the hydrated α/γ phases, and we have previously reported those structures detected simply by using *ex situ* powder XRD experimentation.^13^ Such transitions, which are mirrored in both experimental and theoretical studies in the literature, are thought to improve upon some of the material properties including the crystal order and packing density, leading to higher levels of activity for the electrocatalyst.^23,26,27^ It is also known that the layer contraction, which mostly defines the phase transition, is accompanied by metal cation intercalation, typically K^+^ ions since potassium hydroxide (KOH) is commonly used as alkaline electrolyte.^8,25,28^ With all of that, the improved performance is manifested essentially from an increase in the order of the crystallinity, as well as an observed layer contraction of the structure, which improves the conductivity and hence, the catalytic rate capability of the system.

In addition to the phase, the materials' composition is also believed to be changing during the early catalyst lifetime although the effect on the performance is not well understood. While some studies have demonstrated the inherent instability of NiFe LDH catalysts, there is little emphasis on the cause.^19–21^ Composition changes of mixed-metal hydroxides are also known to occur relatively easily, with many reports of facile Fe^3+^ uptake by LDHs in alkaline media.^28,29^ In the case of synthesized NiFe LDHs with large amounts of iron, there is likely a discrepancy in stability between the less stable ‘guest’ Fe^3+^ and the ‘host’ Ni^2+^, which is stabilized within a brucite-like host structure. It is therefore reasonable to envision that some compositional changes may occur to the Ni^2+^ to Fe^3+^ ratio during OER cycling at high potentials. In this work, we combine electrochemical data with *in situ* and *ex situ* analytical and characterization tools, as well as theoretical calculations to examine in detail the active material before and at various points during and after long-term OER catalysis to shed light on the lack of stability of NiFe LDHs.

## Results

**Fig. 1:**
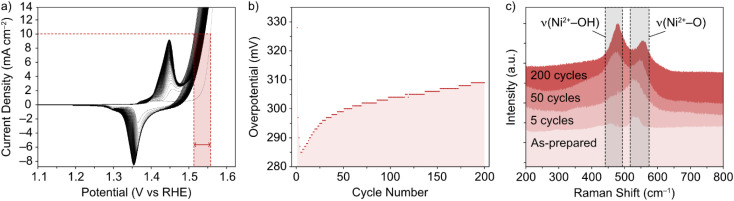
(a) CV of NiFe LDH loaded on nickel foam in 1 M KOH, and (b) resulting overpotential values (±7 mV) for OER as a function of cycle number, through 200 cycles. (c) Corresponding *ex situ* Raman spectra in the selected wavenumber range 200–800 cm^−1^ for NiFe LDH on Ni foam, (from bottom) for the as-prepared catalyst, and after 5, 50 and 200 CV cycles between 1–1.6 V *vs.* RHE at 5 mV s^−1^.

The NiFe LDHs investigated in this work were synthesized by a low temperature method, as previously reported.^30^ The materials exhibited a high degree of crystallinity, which was confirmed by TEM and XRD.^13^ While the general phase behaviour of NiFe LDHs is reasonably well established, the exact effect on the OER performance remains in question, as well as the timescale over which an effect can be seen in the performance. This was the first point addressed and was done so using Raman spectroscopy. To mimic the ‘shut-down’ protocol of an electrolyzer cell (ESI†), CV cycling of the NiFe LDH material was performed in and out of the OER region. The materials' cycling behaviour in this region is demonstrated in Fig. 1a and b and points to the electrocatalytic instability, which is highly reproducible behaviour for NiFe LDH (ESI Fig. S2,† see also previous publication from Tyndall *et al.*^13^).

Raman spectroscopy was used as it is known to give crystallographic phase information of LDHs.^31,32^ The complimentary electrochemical (Fig. 1a and b) and Raman (Fig. 1c) data acquired during the active lifetime of a NiFe LDH catalyst is used to clarify the nature of crystallographic phase transitions in the catalyst and elucidate the reasoning for the decrease in activity over time (Fig. 1b). Studying the Raman spectra in the wavenumber range 200–800 cm^−1^, two significant peaks in the figure at wavenumbers 462 and 527 cm^−1^ indicated the distinctive Raman signal for NiFe LDH.^12^ The former can be attributed to the Ni^2+^–OH band, which is usually present in the range 445–465 cm^−1^ for nickel-based hydroxide crystals.^31,33^ On the other hand, the higher wavenumber band (527 cm^−1^) is commonly assigned to Ni^2+^–O vibrations within a disordered or defective system (although some reports also designate an Fe^3+^–O vibrational contribution in this region for iron-containing hydroxides).^34,35^ This pointed toward the as-prepared sample (Fig. 1c) being largely α-phase hydroxide as opposed to the more ordered γ phase. Studying further *ex situ* spectra and investigating the evolution of the *ν*(Ni^2+^–OH) and *ν*(Ni^2+^–O) bands at various critical points during the catalyst's active lifetime (Fig. 1c), we observed a shift in the relative peak intensities, as well as a push to higher wavenumbers, suggesting a transition towards an increasingly γ-phase material^32^ as proposed by Trześniewski *et al.*^31^ Furthermore, the nature of this peak shift suggests a gradual phase transition during the active lifetime, rather than an instant effect.

**Fig. 2:**
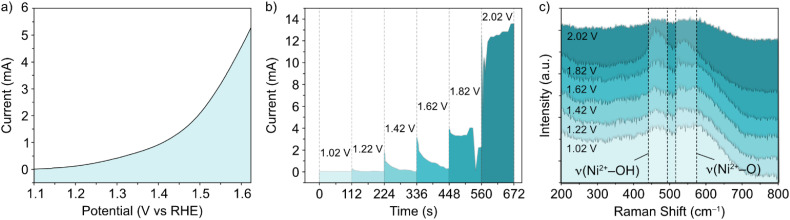
(a) Polarization curve, (b) successive chronoamperometry curves at potentials between 1.02–2.02 V *vs.* RHE, and (c) *in situ* Raman spectra in the selected wavenumber range for NiFe LDH on an indium tin oxide-coated glass substrate, compatible with the *in situ* electrochemical setup.

*In situ* Raman experiments (Fig. 2) suggest that such phase transitions are possible in each potential sweep under these conditions. In the *in situ* cell, a polarization curve (Fig. 2a) can be mimicked by successive chronoamperometry (CA) experiments (Fig. 2b), where selectively increasing potentials are applied at timescales long enough to acquire Raman spectra for each. The results, shown in Fig. 2c, display a similar shift in the main peak intensity ratios during CA scans within the potential window 1–1.8 V *vs.* RHE, compared with *ex situ* data. Beyond this potential limit, the rise in OER activity and concurrent evolution of oxygen bubbles (ESI Fig. S3†) reduces the signal visibility for the *in situ* experimental setup. Regardless, the observable phase transition during the first cycle means that the NiFe LDH must transition between phases with a partial memory effect, and more permanently upon further cycling. These observations could indicate that such phase transitions are a factor in the improved activity of metal hydroxide-type catalysts over *longer* timescales. So, phase considerations alone cannot account for the fluctuating activity.

**Fig. 3:**
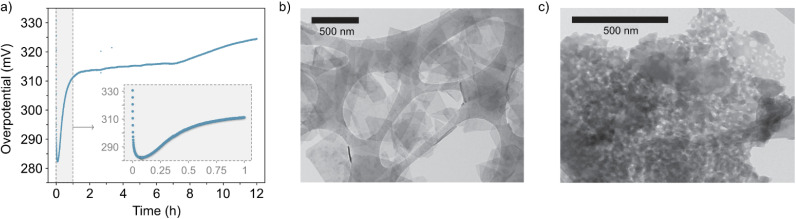
(a) Chronopotentiometry curve of the catalyst at 10 mA cm^−2^ for 12 hours (inset, close-up view of the first hour). (b) and (c) Representative areas of the NiFe LDH samples before and after 50 OER cycles (ESI Fig. S4†), respectively, imaged with STEM.

In addition, the lack of stability in the NiFe LDH material is evident from the increase of the overpotential in the CP experiments (Fig. 3) recorded over a period of 12 hours. Interestingly, scanning transmission electron microscopy (STEM) reveals that the NiFe LDH sample before OER exhibits a layered morphology (Fig. 3b), whereas after OER (Fig. 3c) its morphology changes significantly.

CV and CP experiments (Fig. 1a, b and 3a, respectively) demonstrate the intrinsic instability of NiFe LDH under operating conditions, with impressive performances achieved during early catalyst cycles which deteriorate very rapidly, especially during the first fifty CV cycles (Fig. 1b) or after one hour of CP at 10 mA cm^−2^ (Fig. 3a). This is manifested as a rise in overpotential back towards the initial values. To the best of our knowledge, this apparent loss of activity of NiFe LDH under operando conditions has not been addressed before despite being key to assessing the applicability and potential for industrial compatibility of the material for alkaline electrolyzers.

In order to detect any potential OER-induced compositional degradation within the catalyst after OER, qualitative energy dispersive X-ray (EDX) spectroscopy was carried out *via* scanning transition electron microscopy (STEM) imaging. Firstly, a statistical study was performed over a range of platelets *before* (as synthesized) *versus after* OER (50 cycles in 1 M KOH). Prior to OER cycling, the as-prepared material demonstrated a consistent Ni : Fe ratio at 3 : 1 based on data acquisition over fifteen unique areas of the NiFe LDH sample navigated using STEM (ESI Fig. S5†), which is an expected value based on the synthesis method.^30^ However, after the platelets had been electrocatalytically active, the same level of consistency could not be observed in the Ni : Fe ratio, with most areas displaying a relative depletion in the Fe content with respect to Ni, culminating in an average ratio of 3.8 : 1 (Ni : Fe). In addition, STEM imaging of the post-cycled material (Fig. 3 and ESI Fig. S6†) demonstrates, in some cases, clear morphological change to the active platelets in those areas most affected by the shift in metals composition.

**Fig. 4:**
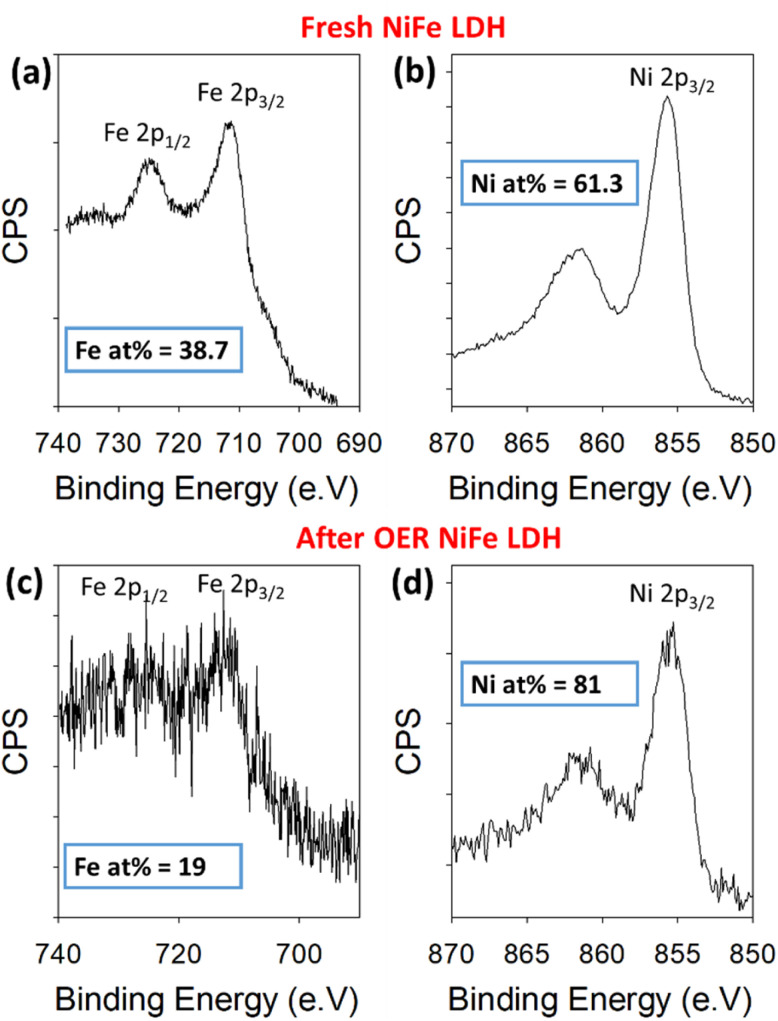
Binding energy XPS spectra for Fe 2p and Ni 2p core levels before cycling (a and b) and after 50 cycles (c and d) within a NiFe LDH catalyst on Au/Ti/SiO_2_/Si.

**Fig. 5:**
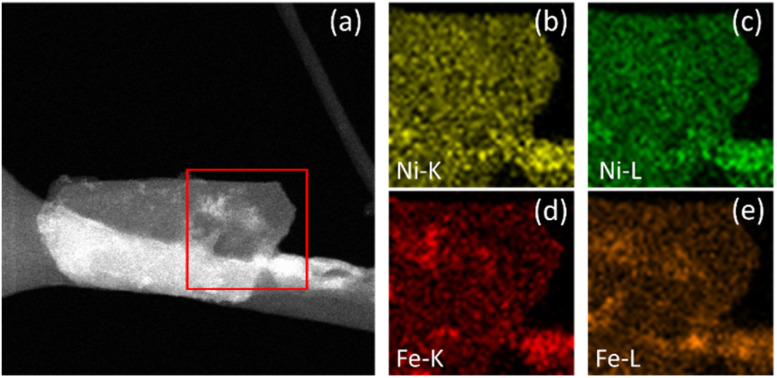
NiFe LDH after OER. STEM image of the area of interest (a) with K and L line EDX maps of Ni (b and c) and Fe (d and e).

To further validate the proposed leaching of Fe from the NiFe LDH, XPS was used to quantify the amount of Ni and Fe in the sample before and after OER. For this, the high-resolution Ni 2p and Fe 2p core levels were measured before and after 50 OER cycles of the catalyst (Fig. 4). The peaks in Fig. 4a and c represent the 2p_1/2_ (725 eV) and 2p_3/2_ (712 eV) regions for Fe^3+^, while those in Fig. 4b and d represent the 2p_3/2_ (855 eV) for Ni^2+^, before and after cycling. Relative atomic percentages were estimated in each case by taking the total integrated counts above the Shirley background within the scan boundaries indicated in Fig. 4, using appropriate relative sensitivity factors. Such estimations are based on assumptions of homogeneous distribution of the Ni and Fe components, both volumetrically and in terms of surface segregation of one component above/below the other. Importantly, the initial atomic % measured for the metals based on the spectra were 61.3% Ni and 38.7% Fe. After cycling, the atomic percentages present a clear change in the material's composition once again. The increase in signal-to-noise ratio at this point is likely a result of signal attenuation through materials on the platelet surface after cycling. These are *ex situ* measurements, after cycling the electrode is rinsed with DI water to avoid KOH deposition, but this is likely not thorough enough to avoid partial coverage. The Ni and Fe atomic percentages at this point are measured at 81% and 19%, respectively, indicating a clear loss of iron from the NiFe LDH structure. This result is consistent with the STEM-EDX image shown in Fig. 5.

Additional STEM-EDX mapping of the affected areas often showed some additional material deposited on the surface of electrocatalytically activated LDH platelets (*e.g.*, the area highlighted in Fig. 5a), demonstrated to be mostly ferric in composition. This is indicated by the increased intensity of specific areas in the Fe-K and Fe-L maps (Fig. 5d and e, respectively) which correlate to the lighter areas in the STEM image. Similar contrast cannot be seen in the equivalent Ni-K and Ni-L maps (Fig. 5b and c, respectively). This is considered as potentially direct evidence of an iron by-product which has been preferentially leached from the LDH structure during catalysis and re-deposited onto the catalyst surface.

**Fig. 6:**
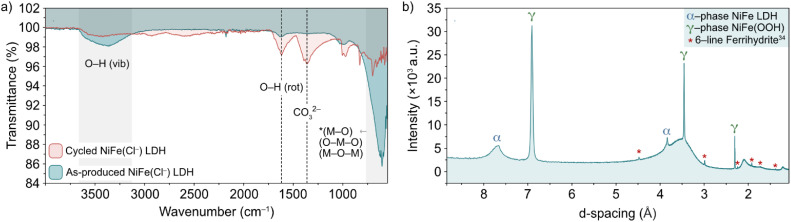
(a) FT-IR spectra of pre-cycled (black line) and post-cycled (red line) Cl^−^-intercalated NiFe LDH and (b) XRD pattern for NiFe LDH after 200 cycles between 1.02–1.62 V *vs.* RHE at 5 mV s^−1^. The α-NiFe LDH and γ-NiFeOOH phases are labelled, as well as a ferrihydrite signal at low *d*-spacing.^1^

The nature of the leached iron from the host LDH structure is best analysed experimentally through post-cycling tests. Through such experimentation, weak signals suggesting a small amount of some ferrihydrite compound were detected through *ex situ* FT-IR spectroscopy and XRD analyses (Fig. 6).^13,36,37^ Prior to FT-IR, an anion exchange step was applied to the as-produced material to replace stable CO_3_^2−^ counter-ions, which are formed as a result of the thermal decomposition of urea during synthesis, with Cl^−^ ions *via* an acid–salt (HCl–NaCl) treatment, in order to remove vibrational features in the wavenumber region of interest (*ca.* 1354 cm^−1^) for this experiment (Fig. 6a, black trace). After cycling, the noticeable emergence of the peak at 1363 cm^−1^ (Fig. 6a, red trace) is likely a result of CO_3_^2−^ re-intercalation (the source of which is likely dissolved atmospheric CO_2_), while the shoulder at 1391 cm^−1^ along with a mostly shielded signal at 1572 cm^−1^ may provide evidence of a small amount of ferrihydrite present after activity.^36^ The presence of ferrihydrite was verified to an extent by XRD analysis performed on the NiFe LDH catalyst after cycling. The diffraction pattern after OER activity (Fig. 6b) not only presented the α/γ dual phase nature of the NiFe LDH catalyst, but additionally showed a clear signal of 6-line ferrihydrite at low *d*-spacing, namely the peaks highlighted at 4.5, 3, 2.25, 1.9, 1.7 and 1.4 Å.^37^ In order to identify a reasonable pathway for the formation of a ferrihydrite by-product *via* iron leaching from the NiFe LDH catalyst, density functional theory (DFT) calculations were carried out.

**Fig. 7:**
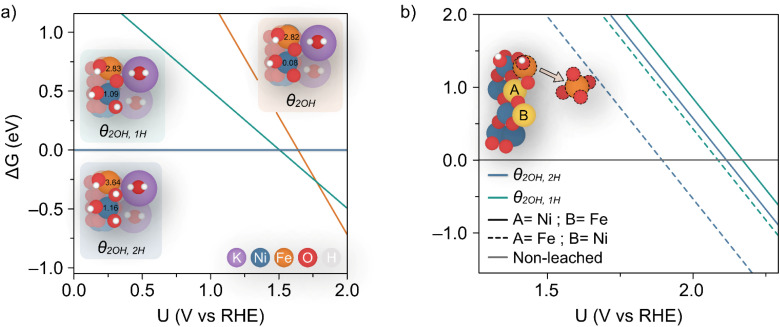
(a) Calculated coverages for the (011̄0) γ-Ni_3_FeO_8_K 2H_2_O surface, with considered coverages depicted as three distinct insets which are predicted most stable from 1–2 V_RHE_. The differing slopes are a result of differing levels of hydrogenation across the three surface coverages. The magnetic moments (in *μ*_B_) for surface Ni and Fe metals are given in the insets. (b) Predicted potentials of Fe(OH) dissolution from differing coverages, since this is the species which leaches first as per ESI Fig. S8 and S9.† The inset shows the two relative positions A and B considered for Fe.

Dionigi *et al.*^8^ provided valuable indications on the nature of the NiFe catalyst using *in situ* experiments along with DFT studies to argue that a semi-reversible phase transition occurs between the α- and γ-NiFe phases. The authors also sampled varying potassium and water ratios intercalating the γ-NiFe structure using an NVT ensemble, which indicated that one K(H_2_O)_2_ unit per Ni_3_FeO_8_ was the most stable configuration. Based on these results, in this work we focused our computational studies on the stability of γ-Ni_3_FeO_8_K·2H_2_O, which recent evidence has indicated to be the material phase that catalyzes the OER.^7^ To investigate stability, we modelled the (011̄0) facet of γ-Ni_3_FeO_8_, since extensive computational studies have pointed towards these sites being responsible for the activity.^38^ For this surface, we first examined the coverage and stability under electrochemical conditions to understand the possible mechanisms of dissolution during the OER. To model the coverage, we enumerated through O and OH adsorbates on the exposed Ni and Fe surface top sites, while also considering the adsorption of H atoms on the bridging O sites. Based on the computed relative Gibbs energies, the most stable coverages between the potential window of interest (*i.e.* 1–2 V_RHE_) correspond to *θ*_2OH,2H_, *θ*_2OH,1H_ and *θ*_2OH_, where this notation denotes the adsorption of two hydroxyl groups on the Ni and Fe top sites, and 2, 1 and 0 H atoms on bridging oxygens, respectively, as depicted in Fig. 7a. To assign the metal oxidation states, we used the magnetic moments obtained from optimised spin polarised calculations. For Fe atoms, magnetic moments of *ca.* 3.6 and 2.8 *μ*_B_ can be attributed to high-spin Fe(iv) and Fe(v), respectively, while for Ni atoms, the values of *ca.* 1.2 and 0.1 *μ*_B_ are characteristic of Ni(iii) and Ni(iv). In doing this, we observed all iron atoms as Fe(iv) and a 1 : 1 ratio of Ni(iii) and Ni(iv) in the coverage *θ*_2OH,2H_. On the other hand, we found that the coverage *θ*_2OH,1H_ sees the surface iron oxidised to Fe(v) with no change to the Ni oxidation state, while the coverage *θ*_2OH_ displays the surface Ni oxidised to Ni(iv). The process of these changes is shown in the insets provided in Fig. 7a. All considered coverages, including those which are not stable in the potential window of interest, are shown in ESI Fig. S7.†

To analyze the potential dissolution mechanisms, we applied the method from Kolpak,^39^ which allows for the analysis of surface reconstructions as a function of pH and potential by treating the Gibbs energy change associated with forming a vacancy on the surface separately to the energy change associated with the reaction of the leached ions to form the solvated species that is most stable for each pH and potential. Under this formalism, the Gibbs energy change associated with the leaching of metal ions can be expressed as:

1 Δ*G*_1_ = *G*_surf−A_ + *μ*_A_ − *G*_surf+A_

where *G*_surf−A_ and *G*_surf+A_ are the Gibbs energy of the surfaces without and with the leached species A, respectively, which has the chemical potential *μ*_A_. The energy change associated with the solvation of the species A is then written as:2
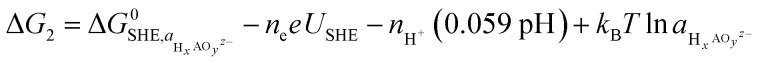
with 
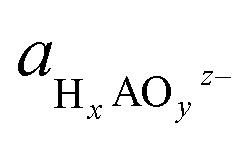
 denoting the activity of the most stable aqueous species for a given applied potential and pH, 
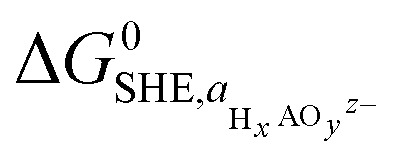
 the experimental Gibbs energy associated to the formation of H_*x*_AO_*y*_^*z*−^ (per A atom) relative to the standard hydrogen electrode,^40,41^ and *n*_e_ and *n*_H^+^_ the number of electrons and protons, respectively, involved in the reaction of the leached species to H_*x*_AO_*y*_^*z*−^. Further details are provided in the ESI.†

Using eqn (1) and (2), we then investigated surface facets resulting from the removal of M(OH)_*x*_ units (M = Fe, Ni; *x* = 1, 2, 3) from coverages which span the potential of interest up to 1.7 V_RHE_, *i.e. θ*_2OH,2H_, and *θ*_2OH,1H_ (Fig. 7a). The results of this analysis are shown in ESI Fig. S8 and S9† for *θ*_2OH,2H_ and *θ*_2OH,1H_, respectively, with Fe dissolution—where Δ*G* < 0—predicted to be favoured over Ni by *ca.* 0.3 V. This can be attributed to the significant change in driving force for dissolution at 1.52 V_RHE_, when the most stable aqueous species becomes [FeO_4_]^2−^. In both coverages, we see the same trend in that Fe(OH) dissolution is favoured over Fe(OH)_2_ since there is overall one more electron transferred in forming [FeO_4_]^2−^ from Fe(OH) as compared to Fe(OH)_2_, which results in a steeper slope and thereby faster intersection with the line Δ*G* = 0. Given that Ni leaching is comparatively much less favoured as the most stable aqueous species is [Ni(OH)_4_]^2−^, in the following we focus on Fe(OH) leaching since it has the largest driving force for dissolution and the same trend is captured across both coverages.

We calculated the predicted potential of Fe leaching where the next nearest neighbour metal in the closest layer is either Ni or Fe, as well as from the coverages *θ*_2OH,2H_, and *θ*_2OH,1H_, as seen in Fig. 7b. Note these coverages were chosen since they are within the potential window of the experiments. Notably, the predicted potential of dissolution varies with respect to the local accumulation of Fe, so that leaching becomes more feasible when Fe atoms agglomerate, in line with experiments shown in Fig. 5.

Although the lowest dissolution potential at 1.9 V_RHE_ predicted by DFT calculations is higher than the observed experimentally (*ca.* 1.55 V_RHE_ in Fig. 1a), our results indicate preferential leaching of Fe over Ni due to ferrate formation. Indeed, recent experiments have reported the use of K_2_FeO_4_ to modulate activity in such a way as to indicate that [FeO_4_]^2−^ is either involved in the OER or in the activation of the catalyst by altering the Ni(ii/iii) redox potential.^42^ Discrepancies in the predicted and experimental dissolution potentials could be attributable to the difficulty in modelling charged species which are represented by the dissolution in Fig. 7b,^43^ as well as the challenge in accurately describing the effect of explicit solvent for this dynamic catalyst.

**Fig. 8:**
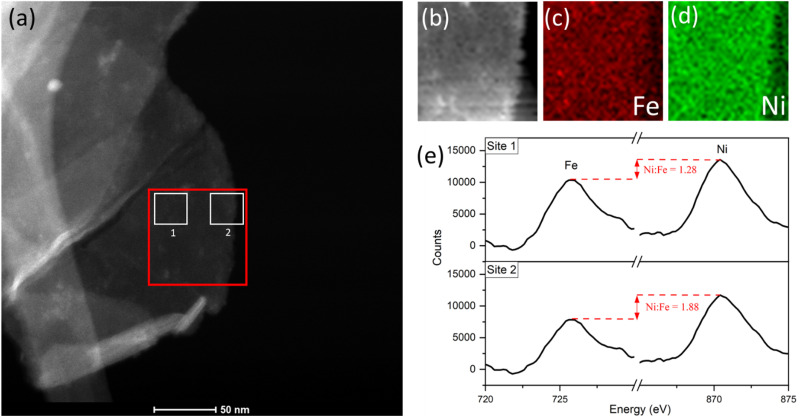
(a) STEM image of platelet after 10 CV cycles, showing the area of interest for EELS mapping analysis (red) and integrated-area spectral analysis (white sites labelled ‘1’ and ‘2’). (b) EELS signal map for the area of interest. (c) Fe and (d) Ni maps. (e) Comparison of the integrated spectra acquired from sites near platelet edge *versus* near the centre, acquired using a common pre-Fe background, with an increase in the Ni : Fe peak intensity ratio from site 1 to 2.

With a reasonable pathway established for the preferential leaching of Fe as a principal degradation route, we posited that post-cycling analysis of the OER active catalyst can identify the degradation as a direct knock-on effect from the water-splitting activity at edge-site surface terminations. This can be done by considering the widely accepted edge-site activity of NiFe LDH and similar catalysts for water-splitting. In theory, if the activity is localized at the edges, then the compositional degradation suggested in this work would start from those areas. To verify this, we performed spatially resolved electron energy loss spectroscopy (EELS) near the edge sites of OER-cycled platelets to observe some contrast in the composition, particularly the iron content near the edge compared to central areas. Data was acquired from an appropriate area (marked out in red, Fig. 8a) of a single platelet after undergoing OER activity on a glassy carbon support, before removal from the support and analysis *via* STEM imaging. Importantly, the platelet must be of uniform thickness in the *x*-direction as it is in this direction which the compositional comparison will be made. Hence, as a preliminary test, the horizontal line profile was acquired across the area of interest (ESI Fig. S10†) to demonstrate uniformity. The uniform thickness was verified by observing the EELS signal map shown in Fig. 8b.

The Fe and Ni EELS signals were mapped spatially as in Fig. 8c and d. Both maps demonstrate the inclusion of the respective metals across the platelet to the edge point. However, it is the Ni signal which demonstrates more homogeneity across the area, with a sharper edge feature compared to the Fe case. The difference in these signals can also be demonstrated more objectively by analysing integrated areas of interest. Namely, by comparing the integrated EELS signals of a site near the centre of the platelet *versus* near the edge (designated as sites ‘1’ and ‘2’ respectively marked out in white in Fig. 8a). The resulting integrated spectra of the sites are shown in Fig. 8e, with a common pre-Fe background removed for consistency. By analyzing the Ni to Fe peak intensity ratio of the respective sites one can notice a clear discrepancy, from Ni : Fe = 1.28 for site 1 to Ni : Fe = 1.88 for site 2. This data is suggestive of spatial inhomogeneity of the leaching effect within the catalyst, meaning there may be more drastic compositional degradation near the platelet edge, during the early catalyst lifetime at the very least. The heterogeneous nature of the EELS data above is echoed in studies for similar OER catalysts, including Ni(OH)_2_. Agoston *et al.*^44^ for example, demonstrate OER-driven compositional and morphological changes for Ni(OH)_2_ catalysts of near-edge structures *via* X-ray absorption near edge structure (XANES) mapping. Together this information may strengthen the argument of edge site activity for LDH catalysts and similar morphologies,^45^ while also suggesting a direct relationship between that activity and the subsequent observable degradation in the material.

## Discussion

With the accumulation of data above, a rounded explanation for the fluctuating activity of NiFe LDH catalysts (Fig. 1a and b and 3a) can be theorized, based principally on a combination of competing crystallographic and compositional changes. The question remains at this point as to what exact effects the respective changes are having on the performance. In terms of phase behaviour, *in situ* experimentation (Fig. 2) provides proof that the potential which accommodates the onset of OER activity comes about beyond the α → γ phase transition, which is believed to be semi-reversible in nature (Fig. 2c). So, knowing that such a change in phase will improve the performance, can this be characterized as a short term or long-term effect *i.e.*, is the phase behaviour causing the sharp fluctuations in NiFe LDH performance during the first 50 cycles (Fig. 1b), or not?

To shed light on this point, we compared the early cycling behaviour of NiFe LDH with that of a pure Ni metal catalyst, since Ni(OH) catalysts are known to experience similar phase behaviour as described by the Bode scheme,^24^ and without the possibility of any preferential leaching effects on the performance. Compared to the mixed-metal LDH case, pure Ni catalysts experienced no fluctuation within the initial 50 cycles, but rather a steady decline of the overpotential (ESI Fig. S11†), signifying gradual performance improvement, presumably due to the gradual shift in the crystal phase. With that, we posited that the inherent instability of NiFe LDH catalysts for alkaline water-splitting during the early active lifetime is a result of compositional degradation, namely the preferential leaching of Fe. The Ni : Fe ratio is optimized at a value just above that of the as-prepared material, meaning that at the very early stages of activity, the loss of Fe will cause the Ni : Fe ratio (and hence performance) to approach, and then surpass the optimum value for OER before reaching a point of relative stability, for example after roughly 50 CV cycles (Fig. 1b) and 1 hour of constant current (Fig. 3a). An important additional consideration here is that of Fe re-deposition. Small amounts of Fe species present in the KOH are known to deposit on Ni and NiFe catalyst surfaces, effecting the composition and in some cases hence, the performance.^28,46^ Recently Bao *et al.*^47^ reported on the re-deposition of residual Fe from KOH electrolytes on NiFe catalyst surfaces, occurring in the ‘recovery’ potential range (*i.e.* the potential range below the NiFe(OH)_2_/NiFeOOH redox couple in Fig. 1a) where deposition is favourable. The authors reached this conclusion using similar experimental approach to the presented work, namely a combination of continuous and intermittent electrochemical analyses, as well as XRD, Raman and XPS analysis for insights on composition. Relating back to the presented work, such a re-deposition affect may be the reason for the sharper deactivation observed in CP curves (*e.g.*, Fig. 3a) compared to CV cycling curves over similar timescales (*e.g.*, Fig. 1b), as the former spends no time in the recovery range. With that, if quantitative compositional analysis is to be performed in the future, CP experiments may present less ambiguity in this respect.

Beyond the point of initial deactivation (∼50 CV cycles per 1 h CP for this work), the long-term activity continues to gradually fade. One consideration to the cause of this is the gradual and continued growth of a hydrous oxide surface layer which, after reaching a critical thickness, can act to inhibit the catalyst in terms of surface kinetics.^48,49^ Lyons *et al.*^50^ reported that the redox switching kinetics within the hydrous oxide layer is promising in thin layers, but may be inhibiting upon further growth, in terms of the diffusion of reaction intermediates through the layer. The ongoing, sequential growth of the layer, signified by the continued peak intensity growth of the Ni^2+^/Ni^3+^ redox couple (ESI Fig. S12†), may be another contributing factor to the subtle loss of activity observable at longer active timescales of highly crystalline NiFe LDH catalysts.

## Conclusions

Overall, this work demonstrates a clear compositional degradation effect within NiFe LDH electrocatalyst during the active lifetime in alkaline water electrolyzer cells which has a direct effect on the short-term performance. The compositional changes in question involve the preferential leaching of the Fe metal component with respect to Ni, from the octahedral sites within the LDH structure. This is suggested by a combination of quantitative EDX (ESI Fig. S5 and S6†) and XPS surveys (Fig. 4), while spatially resolved EELS mapping reveals a higher proportion of the leaching effect near the active edge sites of the platelets. This may be an indication of the direct link between OER activity and compositional degradation. It is no surprise then that such drastic fluctuations in OER activity are observed for this choice of catalyst when such a change is occurring around the active areas. DFT calculations have identified a pathway for the removal and dissolution of Fe from various OER relevant surface terminations. Calculations determine that the removal of surface Fe(OH) units to ultimately form [FeO_4_]^2−^ ions are thermodynamically favoured under OER conditions. Subsequent washing and drying steps would likely lead to the precipitation of the ferrihydrite observed in post-cycling analysis *via* FT-IR and XRD (Fig. 6). Despite crystallographic phase changes influencing the catalyst performance over longer timescales, we conclude that the activity fluctuation is the result of rapidly changing Ni : Fe ratios at the catalytically active edge sites.

In terms of future impact based on the results of this research, a more precise synthetic approach is likely necessary in terms of host–guest engineering of the Ni and Fe metal centres, in combination with approaches such as platelet size engineering and composite preparation to optimize catalyst output. In addition, the future of OER catalysis research should have more emphasis on meaningful, practical reporting including electrochemical testing on timescales which reflect the practical applications. In this way, important characteristics such as catalyst compositional degradation, as reported here, may be determined earlier to allow for this information to be utilised to fabricate better OER catalysts.

## Experimental section

### NiFe LDH synthesis

Ni(NO_3_)_2_·6H2O, urea, Fe(NO_3_)_3_·9H_2_O and TEA were made into a 200 ml solution using DI water such that their respective concentrations were 7.5 mM, 2.5 mM, 17.5 mM and 10 mM. The resulting solution was stirred using a magnetic stirring bar at 150 rpm at room temperature for 24 h. The reaction mixture was heated with stirring under reflux to 100 °C in a mineral oil bath for 48 h. Afterwards, the system was allowed to cool naturally to room temperature. The dispersion was centrifuged at 3000 rpm for 10 minutes to separate the precipitate from the solvent. The material was then washed three times with DI water by shaking and then centrifuging to separate. The clean material was then re-dispersed in IPA with an additional two washing steps. Platelets were then tip-sonicated using a Fischer Scientific Sonic Dismembrator Ultrasonic Processor at 40% power for 1 hour (with Fisher Scientific Isotemp refrigerated bath circulator cooling system held at 5 °C).

### Anion exchange

100 mg of the as-prepared platelets were added to a 100 ml solution of 1 M NaCl + 3.3 mM HCl (for Cl^−^ anion exchange). The resulting solution was bubbled under N_2_ gas for 2 h and stirred for a further 24 h (150 rpm), before being centrifuged at 3000 rpm for 5 min and the sediments collected and washed three times with DI water by centrifugation, as before.

### Electrode preparation

Electrodes for OER testing were prepared by spraying NiFe LDH component onto a choice of substrate, depending on the characterization, using a USI Prism Ultracoat 300 spray tool with the substrate kept at 100 °C. A flow rate of 0.5 ml min^−1^ was used and mass loading was monitored using a Sartorius SE2 ultra-microbalance. The choice of substrates were Ni foam for *ex situ* Raman, indium tin oxide for *in situ* Raman, glassy carbon for STEM-EDX/EELS, FT-IR and XRD, and finally Au/Ti/SiO_2_/Si for XPS analyses.

### Characterization

STEM-EDX was performed using a JEOL 2100 LaB TEM microscope equipped with an Oxford X-Max 800 mm^2^ EDX detector from Oxford Instruments. STEM-EELS was carried out using an uncorrected FEI TITAN microscope operating at 300 kV. The microscope was equipped with the Gatan Imaging Filter (Gatan Inc., CA, USA). Typically, EELS spectra were acquired with a dispersion of 0.1 eV per channel with an energy resolution of ∼1 eV (FWHM of the zero-loss peak) and an entrance aperture of 2 mm on the spectrometer. FT-IR spectra were acquired using a PerkinElmer Spectrum 100 FT-IR spectrometer in the range 4000–550 cm^−1^. Powder XRD was performed using a Bruker Advance Powder X-ray diffractometer with a molybdenum K-α emission source in the Bragg–Brentano configuration. XPS measurements were obtained using an Omicron EA 125 Energy Analyser with a monochromated Al K-α source at 1486.7 eV, where the binding energy was calibrated to the C 1s peak. High resolution core level component XPS scans were obtained with a pass energy of 20 eV, high magnification mode, entrance and exit slits of 6 mm and 3 mm respectively giving an overall total resolution of 0.6 eV with an instrument resolution of 0.46 eV. Raman spectra for this work were acquired using a WITec Alpha 300R with a 532 nm excitation source at 1 mW with 1800 lines per mm spectral grating.

### Electrochemical testing

All CV, LSV, CA and CP testing was performed in a conventional three-electrode setup with a Pt counter electrode, Ag/AgCl reference electrode (3.5 M KCl filling solution) and 1 M aqueous KOH solution (J. T. Baker) as the electrolyte, using a BioLogic VMP 300 potentiostat. Voltammograms were performed in the range 0–1 V (*vs.* Ag/AgCl) at 5 mV s^−1^ scan rate. The *in situ* experimental setup involved the use of a magnetic mount Raman electrochemical flow cell (Redox. Me) with a sapphire window with a Gamry Reference 600 potentiostat. The potentials measured *vs.* Ag/AgCl were mathematically converted to potential *vs.* reference hydrogen electrode (RHE) using the following equation:
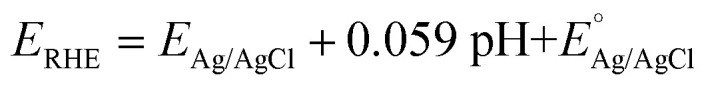
where 
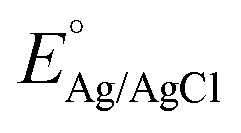
 is the saturated *E*_Ag/AgCl_ electrode potential equal to 0.197 V at 25 °C.

### Computational methods

DFT calculations reported in this work were performed at the PBE + U level^51^ using the Vienna *Ab initio* Simulation Package (VASP) code, version 5.4.4.^52,53^ Core electrons of Ni, Fe, O, K and H atoms were described using projected-augmented wave (PAW) pseudopotentials^54^ and a kinetic energy cut-off for the plane waves of 500 eV. For Fe and Ni metals, on-site corrections for localized 3d electrons were included through Dudarev's framework^55^ using effective Hubbard values of 4.3 ^56^ and 6.2 eV,^57^ respectively. *Γ*-Centered calculations with a 1 × 4 × 3 *k*-point grid were used, where the *x*-direction denotes the direction parallel to the vacuum. Electronic steps were converged to 10^−6^ eV and structures were deemed converged when all forces on atoms were less than 0.02 eV Å^−1^ and self-consistent electronic energies were converged to 10^−6^ eV. These γ-NiFe surfaces were derived from those provided from the work of Dionigi *et al.*,^7^ found under ‘gamma-NiFe-LDH. cif’ within the zip file found by following the hyperlink ‘ESI CIF Files’.† The bulk structure was optimized with *Γ*-centred *k*-point grid spacing of 2 × 4 × 3, and a plane wave cut-off of 520 eV. The cell was fully relaxed with the interlayer distance converging to 7.45 Å, and this structure was then used to create the (011̄0) facet. Calculations were carried out using the Methfessel-Paxton of order 1 with a Gaussian smearing of 0.2 eV. The valence configurations for Ni, Fe, K, O and H atoms were 3d^8^4s^2^ for Ni (10 valence electrons), 3d^6^4s^2^ for Fe (8 valence electrons), 3s^2^3p^6^4s^1^ for K (9 valence electrons), 2s^2^2p^4^ for O (6 valence electrons) and 1s for H (1 valence electron).

Binding energies were calculated using the computational hydrogen electrode model assuming an equilibrium between H_2_ and protons and electrons at 0 V.^58^ Gibbs corrections to the electronic energies were computed for each individual adsorbate at the experimental conditions of 300 K and 1 atm. These include zero-point energy (ZPE), and vibrational entropic and enthalpic contributions calculated using the harmonic approximation provided by the Thermochemistry python module within the Atomic Simulation Environment (ASE) library,^59^ according to the following equation:Δ*G*_i_ = Δ*E*_i_ + ΔZPE_i_ − *T*Δ*S*_i_where index i denotes an adsorbate.

Gibbs corrections for gaseous H_2_ and H_2_O molecules were calculated using the ideal gas approximation. For water, these corrections were computed at 298.15 K and 0.03 atm since the liquid and gaseous phases are in equilibrium at these conditions.^60^ The corrections for the gas phase species and adsorbates are summarized in Table S1.†

## Author contributions

D. T., M. P. B., and V. N. took part in developing the concept and structuring of the project and experimental approach. Synthesis and anion exchange of NiFe LDH platelets was carried out by D. T. Electrochemical testing including *in situ* measurements were performed by D. T. with assistance and analytical input from M. P. B. *In situ* Raman spectra were acquired by N. M. and *ex situ* spectra by D. T. STEM/EDX and EELS analyses, including mapping and statistical data acquisition was performed by A. R. XPS surveys were acquired by L. G. under the supervision of C. M., and fitted by M. P. B., who also performed subsequent atomic ratio calculations. FT-IR was performed by D. T. XRD was analysed by D. T. Finally, DFT calculations and theoretical report were performed and presented by M. G. M. and M. C. This paper was written by D. T. with assistance from M. P. B. and V. N. All authors contributed to the manuscript.

## Conflicts of interest

There are no conflicts of interest to declare.

## Supplementary Material

TA-011-D2TA07261K-s001
