# Supplementary material for: Demonstrating the source of inherent instability in NiFe LDH-based OER electrocatalysts
Source: J Mater Chem A Mater. 2023 Jan 17;11(8):4067–77. doi: 10.1039/d2ta07261k (PMC9942694; doi:10.1039/d2ta07261k)
Supplement: TA-011-D2TA07261K-s001 [file TA-011-D2TA07261K-s001.pdf]

Electronic Supplementary Material (ESI) for Journal of Materials Chemistry A.
This journal is © The Royal Society of Chemistry 2023

Supplementary Information

Demonstrating the Source of Inherent Instability in NiFe LDH-Based OER Electrocatalysts

Daire Tyndall[1,2], Michael Craig[1,2], Lee Gannon[2,3], Cormac McGuinness[2,3], Niall McEvoy[1,2], Ahin Roy[4], Max García-Melchor[1,2], Michelle P. Browne[1,2,5] and Valeria Nicolosi[1,2,6*]

[1]School of Chemistry, Trinity College Dublin, College Green, Dublin 2, Ireland
[2]CRANN and AMBER Research Centres, Trinity College Dublin, College Green, Dublin 2, Ireland
[3]School of Physics, Trinity College Dublin, College Green, Dublin 2, Ireland
[4]Materials Science Centre, Indian Institute of Technology Kharagpur, West Bengal, India – 721302
[5]Helmholtz-Zentrum Berlin für Materialien und Energie, 14109 Berlin, Germany
[6]I-Form Research, Trinity College Dublin

*Email: **nicolov@tcd.ie**

Electrochemical testing approach for alkaline electrolyser cells

As this technology will be implemented as an intermittent source of energy, the performance of the cell before and after 'shut-down' is of critical importance. As, if performance is lost when the cell is 'turned back on' this is a rise for concern. This work will principally use cyclic voltammetry (CV) testing to periodically 'shut-down' the cell and 'power on' again while tracking the changes within the active material. Additionally, chronoamperometry (CA) and chronopotentiometry (CP) experiments will be applied where appropriate. CP experiments are performed by fixing the input current passing through the cell while monitoring the cell potential as a function of time, which gives an insight into an alternative mode of cell activity compared to CV. Similarly, a CA experiment applies a constant voltage to the cell while the cell current is monitored. This mode is used here to mimic a potential cycle on timescales which allows for simultaneous monitoring of electrochemical activity and *in-situ* spectroscopy of the active material.

Thermodynamic analysis of leaching

To determine the Gibbs energy associated to metal leaching, ΔG, we applied the approach reported by Kolpak et al. which conceptually splits the total Gibbs energy of formation of the leached surface in two parts.[1] The first one involves the energy change in going from the clean to the leached surface, ΔG_1

$$\Delta G_1 = G_{surf-A} + \mu_A - G_{surf+A} \tag{1}$$

where G_{surf-A} and G_{surf+A} are the Gibbs energy of the surfaces without and with the leached species A, respectively, which has the chemical potential μ_A. This latter term is calculated using the corresponding metal bulk energy per atom, for which we do not calculate Gibbs corrections, and the Gibbs energy of H_2O and H_2. For example, for $A =$ Fe(OH), μ_A is computed as the potential energy per atom of bulk Fe and the Gibbs energy of OH, calculated as $G_{H2O} - G_{H2}$.

The second contribution to the Gibbs energy of formation of the leached surface, ΔG_2, corresponds to the energy required in going from the leached substance A to its solvated form, H_xAOy^{z-}, over the range of potentials at the experimental $pH = 14$

$$\Delta G_2 = \mu_{H_xAOy^{z-}} - \mu_A - \sum_i n_i \mu_i \tag{2}$$

where $i = e^-, H^+, H_2O$. We relate then chemical potentials μ_i to their standard states μ_i^o using

$$\mu_{H_xAOy^{z-}} = \mu^o_{H_xAOy^{z-}} + k_B T ln a_{H_xAOy^{z-}} \tag{3}$$

$$\mu_{H^+} = \mu^o_{H^+} + k_B T ln a_{H^+} \tag{4}$$

$$\mu_{e^-} = \mu^o_{e^-} - eU_{SHE} \tag{5}$$

$$\mu_{H_2O} \approx \mu^o_{H_2O} \tag{6}$$

Upon substitution into the Equation 2 for ΔG_2, and using $\Delta G^{\circ}_{SHE} = \mu^{o}_{H_xAOy^{z-}} + n_{H^+}\mu^{o}_{H^+} + n_{e^-}\mu^{o}_{e^-} - \mu^{o}_{A} - n_{H_2O}\mu^{o}_{H_2O}$, we can calculate our energies of leaching relative to A with respect to the standard hydrogen electrode (SHE) as:

$$\Delta G_2 = \Delta G^{\circ}_{SHE} - n_e(eU_{SHE}) - n_{H^+}(0.059pH) + k_BTlna_{H_xAOy^{z-}} \quad (7)$$

where ΔG°_{SHE} is the standard hydrogen electrode Gibbs energy per leached substance obtained from experiments.[2–4]

Supplementary Figures

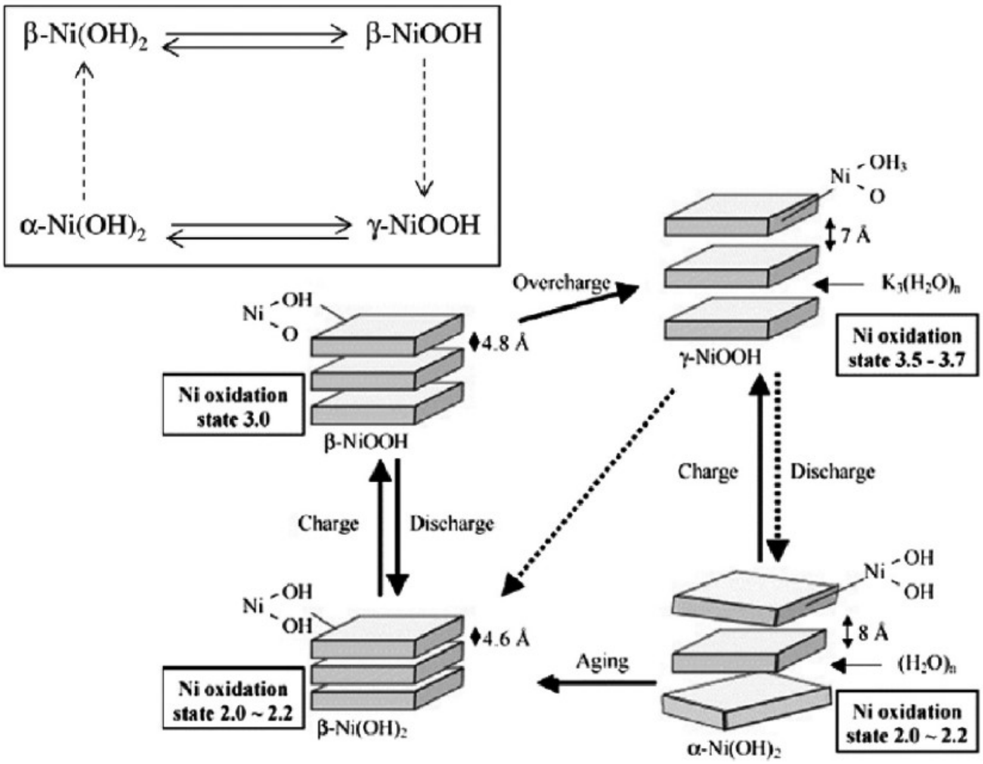

Figure 1: The Bode diagram, a schematic representation of the established phase transitions for a nickel hydroxide OER catalyst. [2]

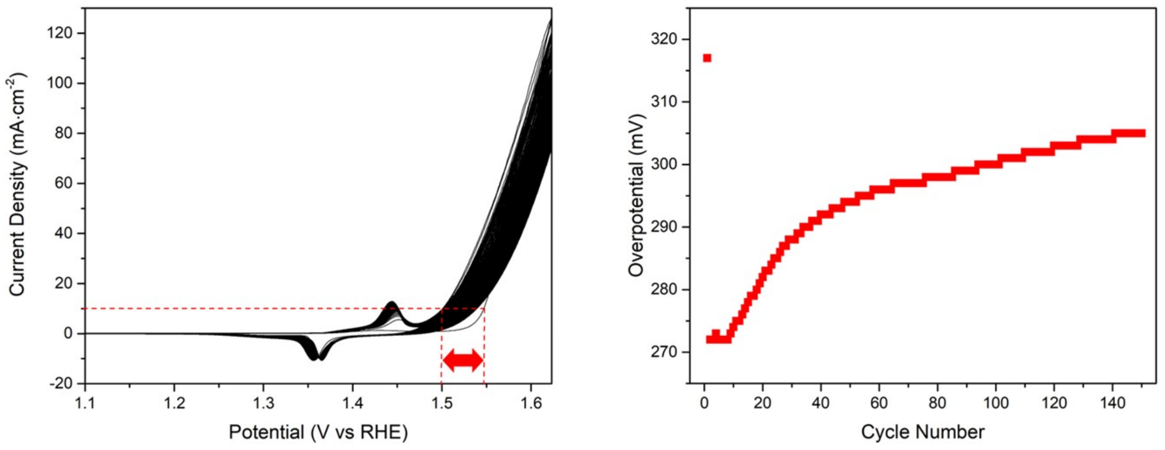

Figure 2: (a) CV of NiFe LDH loaded on nickel foam in 1 M KOH, and (b) resulting overpotential values for OER as a function of cycle number, through 150 cycles. This data compliments the cycling behavior demonstrated in Figure 2 of the main text.

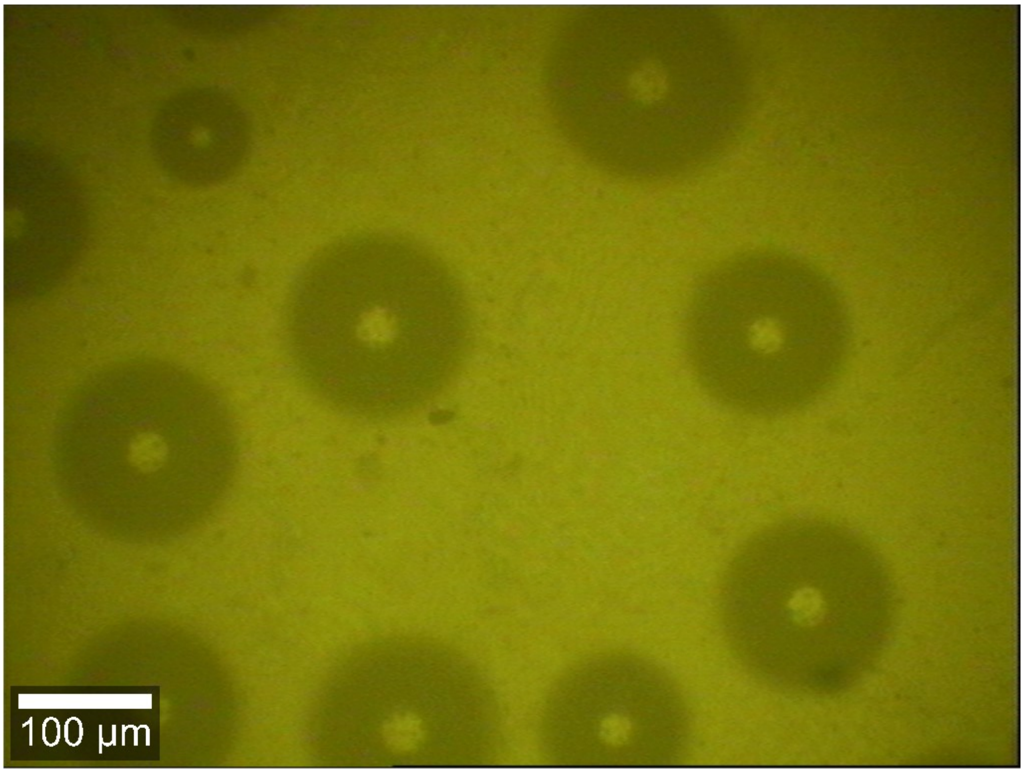

Figure 3: Digital micrograph capturing the evolution of large oxygen bubbles on the surface of a NiFe LDH-loaded ITO electrode within an in-situ alkaline water electrolyser setup.

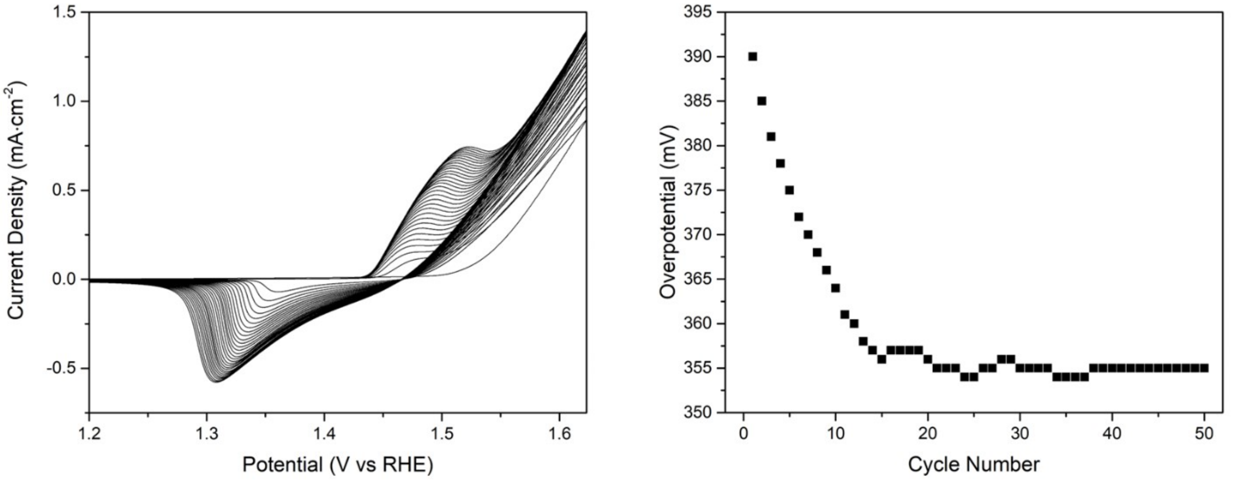

Figure 4: (a) CV and (b) overpotential cycling curve at 1 mA·cm^{-2} onset potential, of NiFe LDH catalyst on GC through 50 cycles.

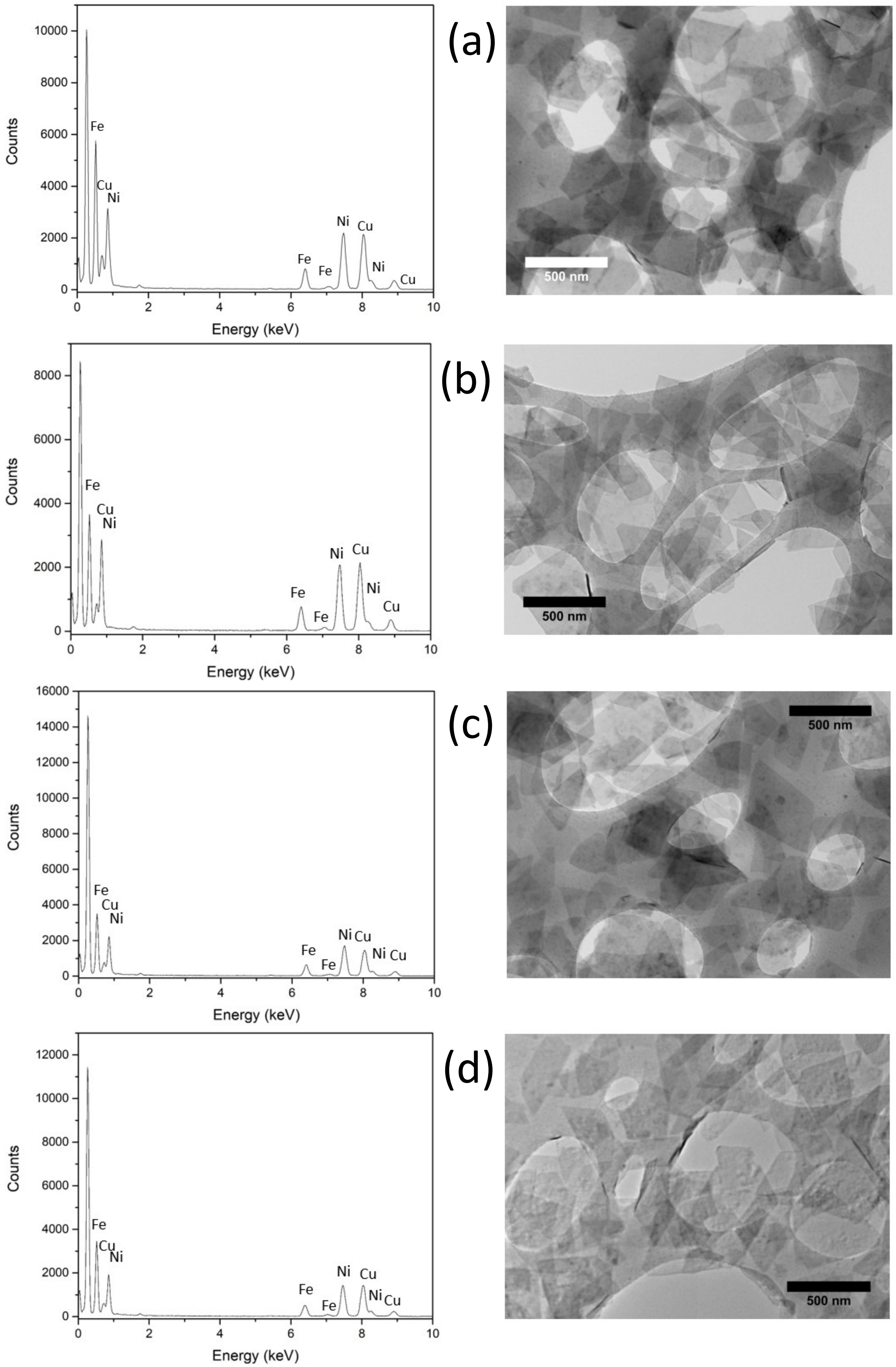
(a)
(b)
(c)
(d)
Counts
Energy (keV)
Fe
Cu
Ni
500 nm

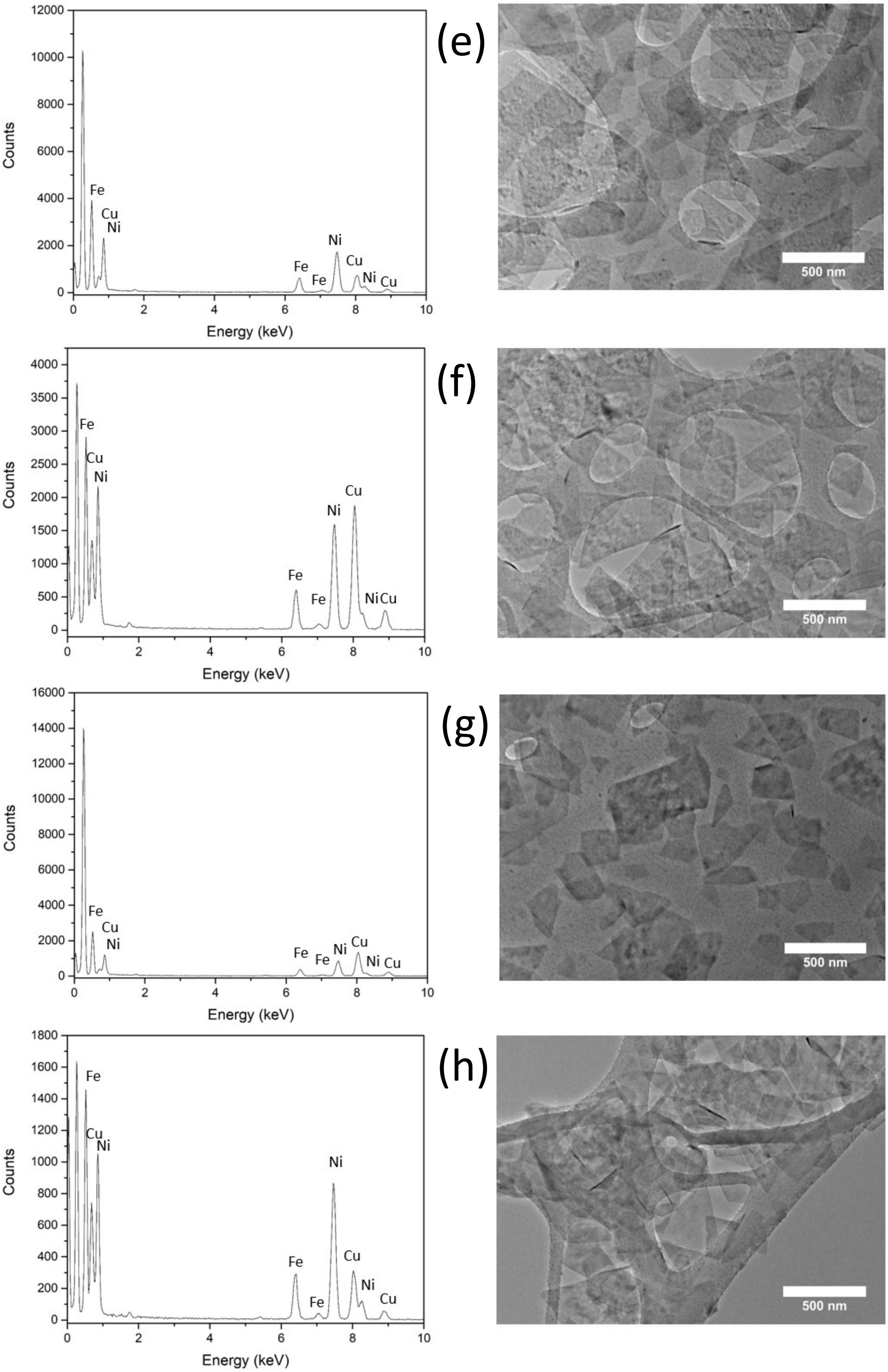
(e)
Counts
Energy (keV)
Fe
Cu
Ni
Fe
Fe
Ni
Cu
Ni
Cu
500 nm
(f)
Counts
Energy (keV)
Fe
Cu
Ni
Fe
Fe
Ni
Cu
Ni Cu
500 nm
(g)
Counts
Energy (keV)
Fe
Cu
Ni
Fe
Fe
Ni
Cu
Ni
Cu
500 nm
(h)
Counts
Energy (keV)
Fe
Cu
Ni
Fe
Fe
Ni
Cu
Ni
Cu
500 nm

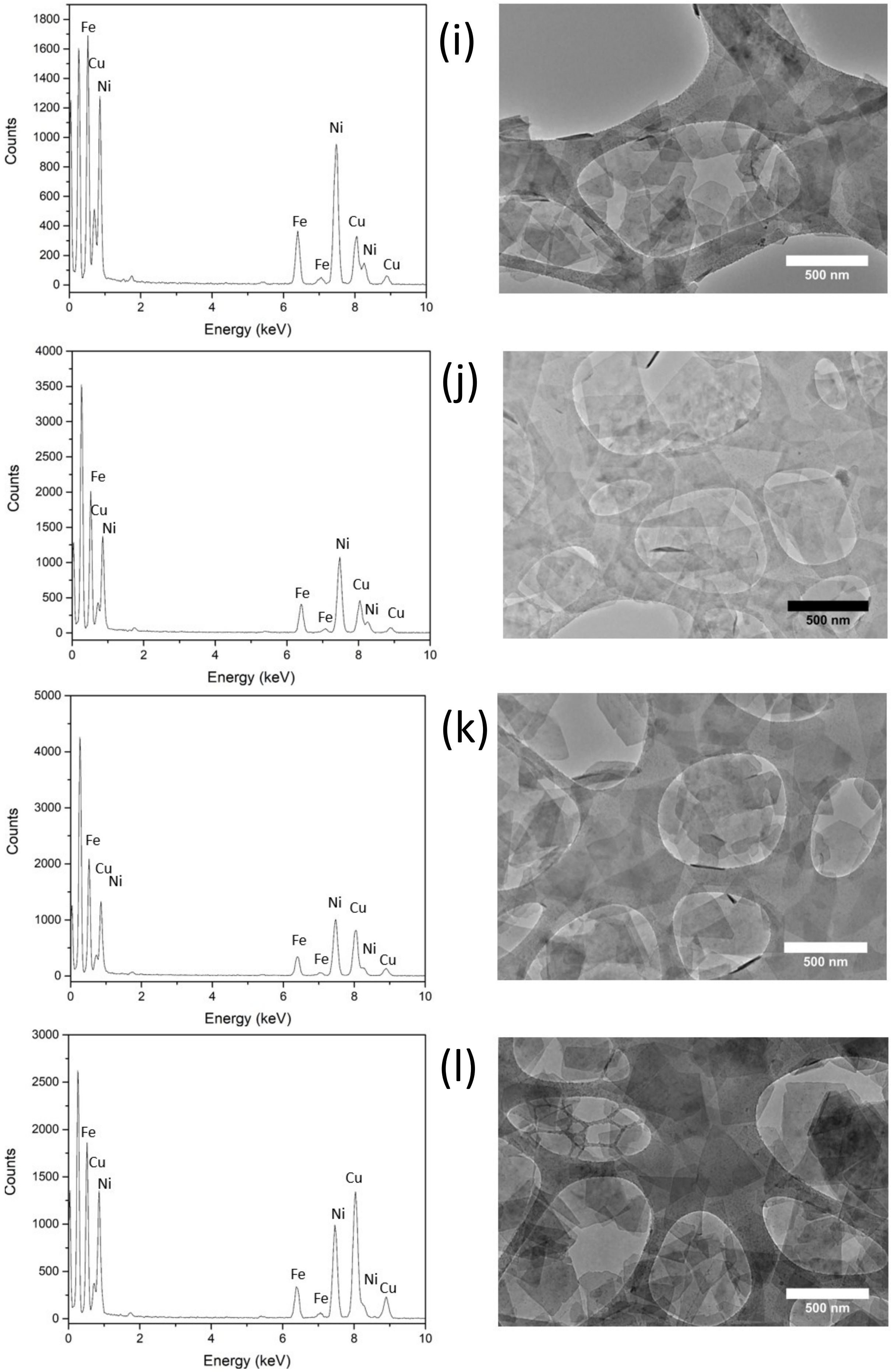

(i)
(j)
(k)
(l)
Counts
Energy (keV)
Fe
Cu
Ni
500 nm

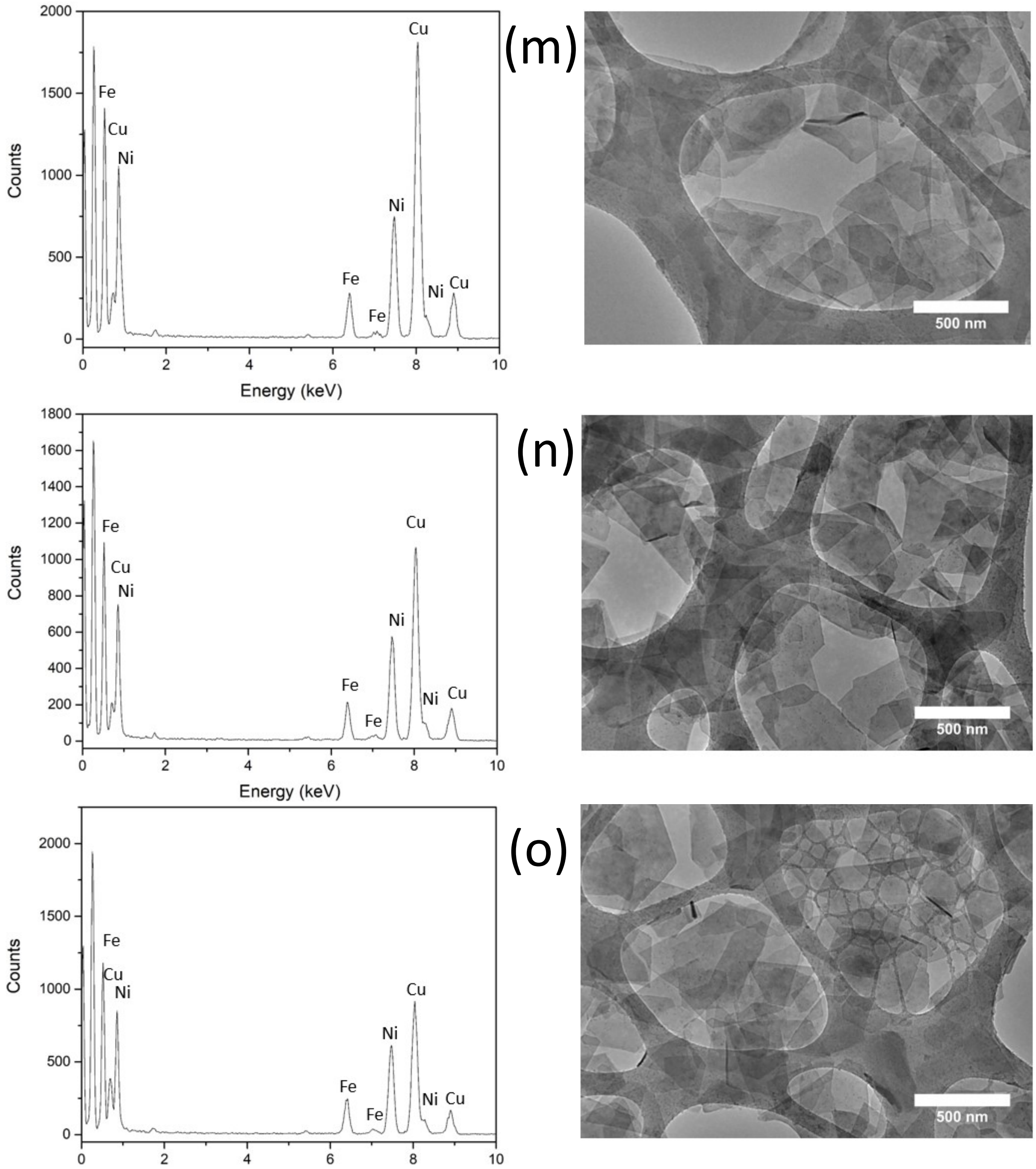

Figure 5: EDX spectra and accompanying STEM images for the pre-cycled NiFe LDH.

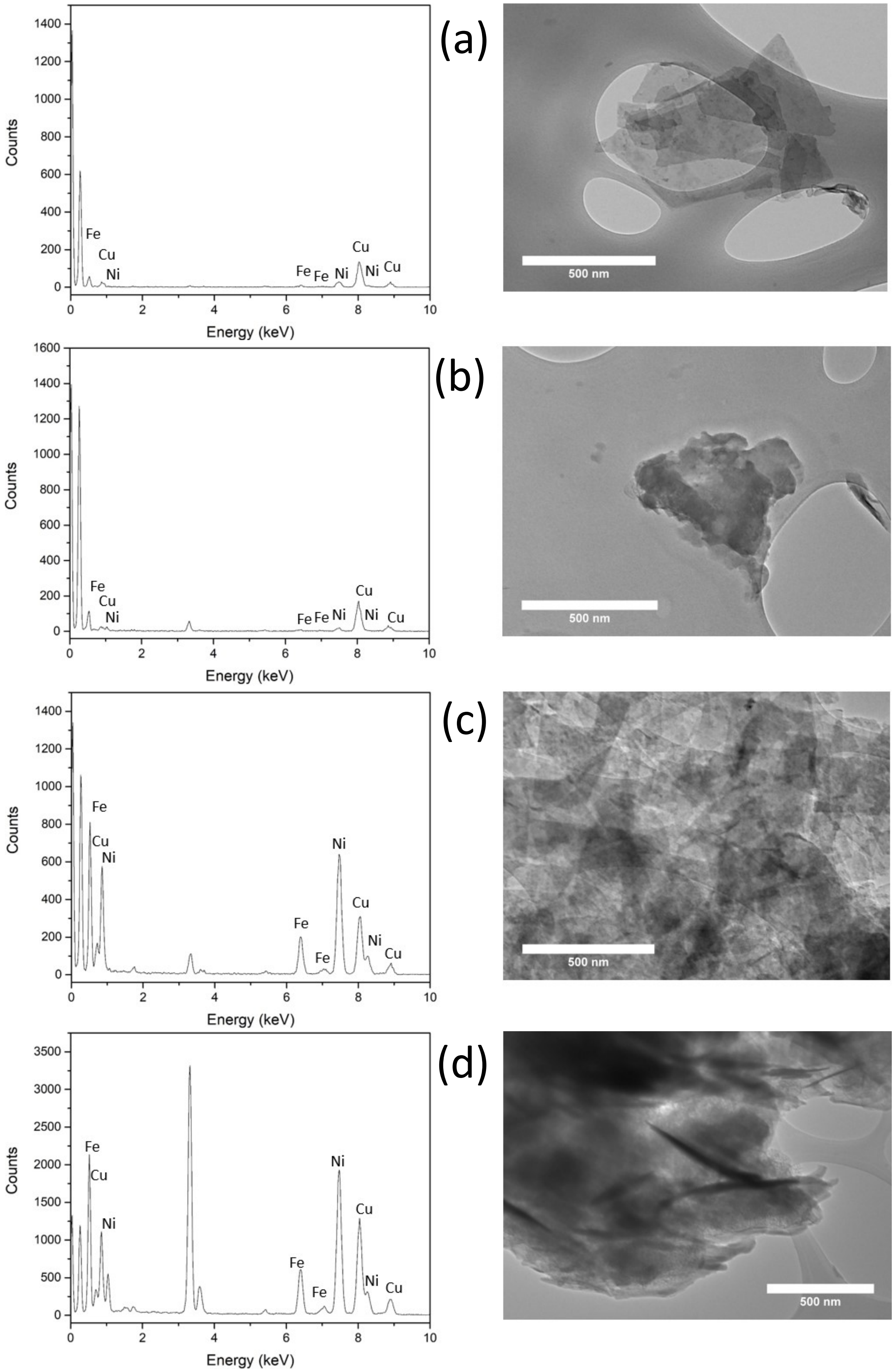
(a)
(b)
(c)
(d)
Counts
Energy (keV)
Fe
Cu
Ni
500 nm

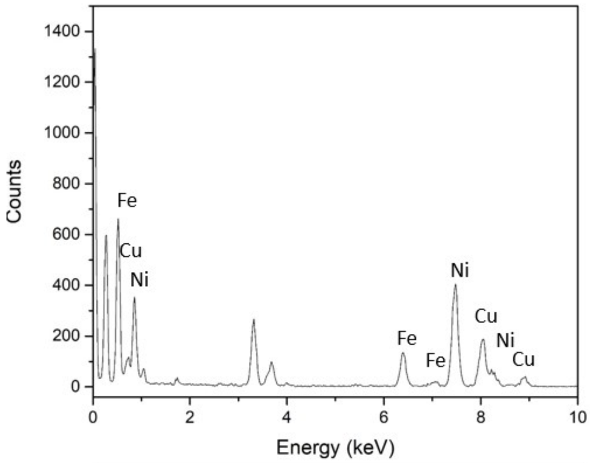
Counts
Energy (keV)
Fe
Cu
Ni
Fe
Fe
Ni
Cu
Ni
Cu

(e)

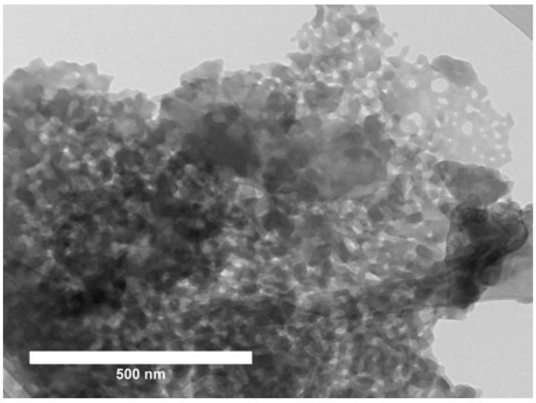
500 nm

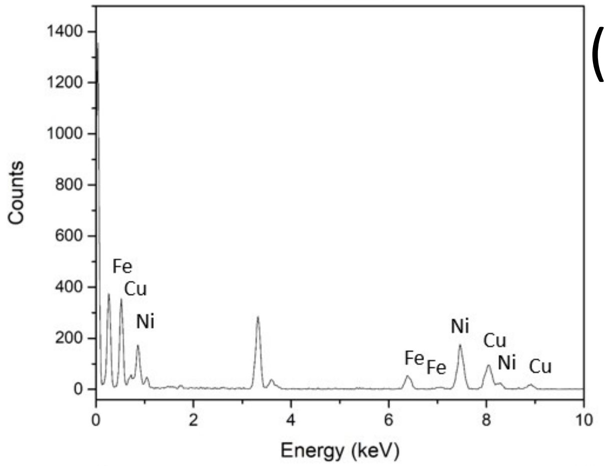
Counts
Energy (keV)
Fe
Cu
Ni
Fe
Fe
Ni
Cu
Ni
Cu

(f)

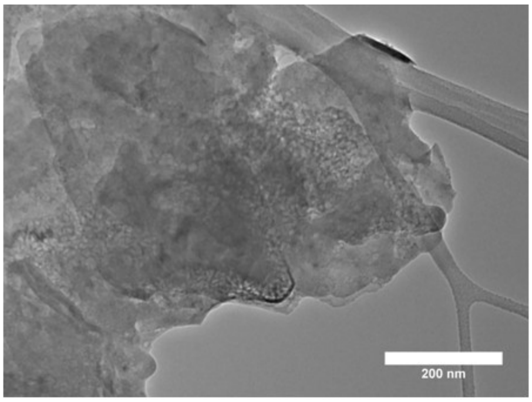
200 nm

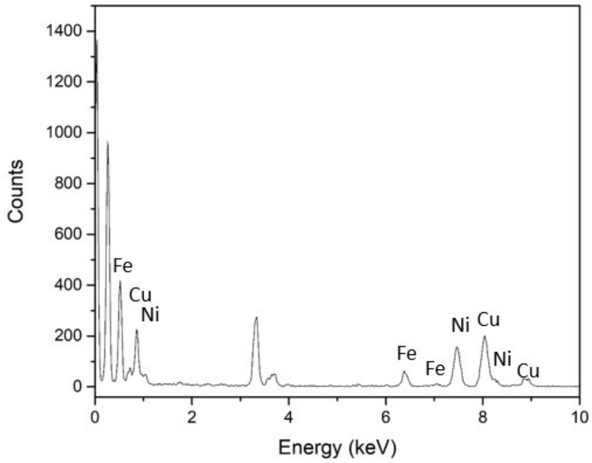
Counts
Energy (keV)
Fe
Cu
Ni
Fe
Fe
Ni
Cu
Ni
Cu

(h)

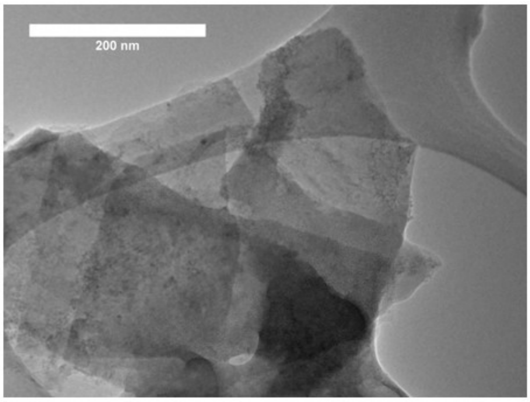
200 nm

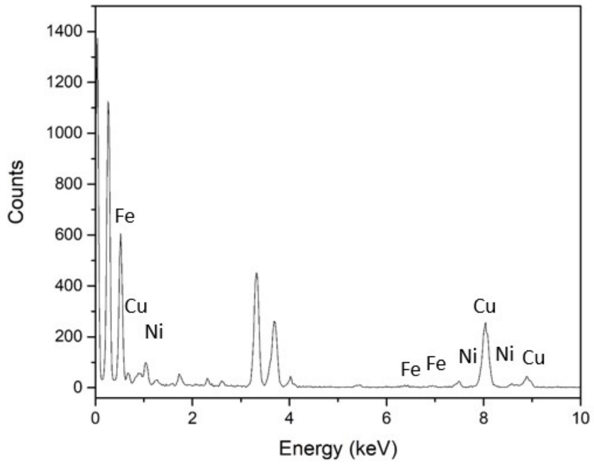
Counts
Energy (keV)
Fe
Cu
Ni
Fe
Fe
Ni
Ni
Cu
Cu

(h)

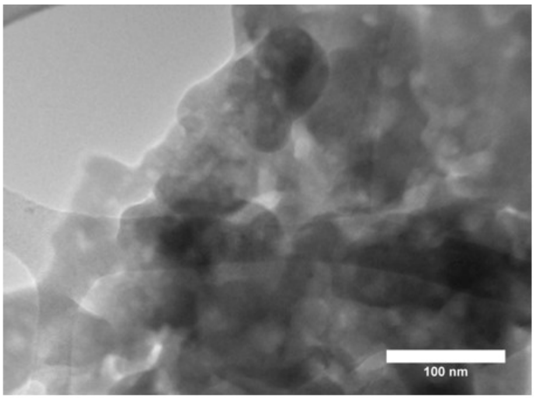
100 nm

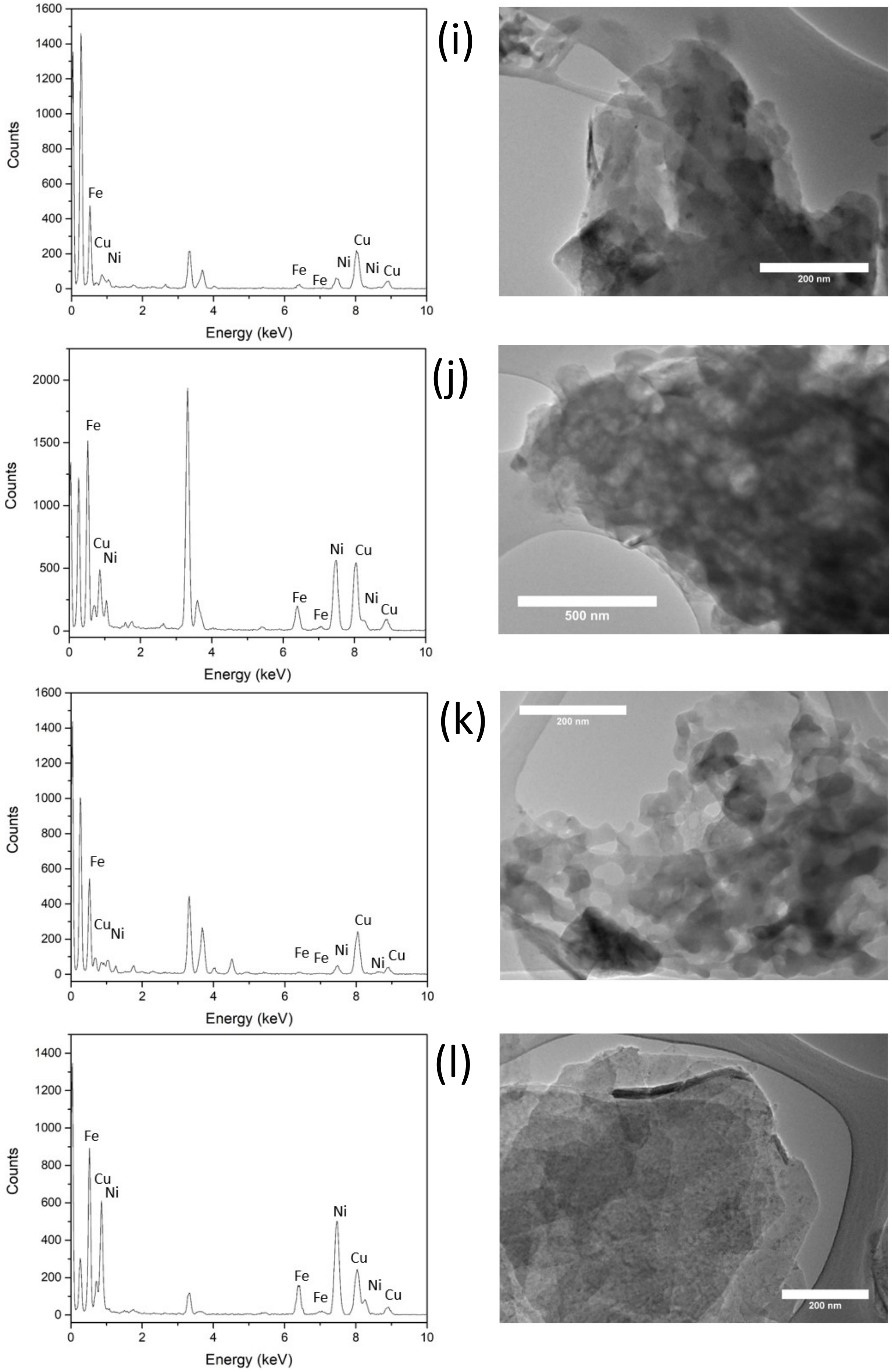
(i)
Counts
Energy (keV)
Fe
Cu
Ni
Fe
Fe
Ni
Cu
Ni
Cu
200 nm
(j)
Counts
Energy (keV)
Fe
Cu
Ni
Fe
Fe
Ni
Cu
Ni
Cu
500 nm
(k)
Counts
Energy (keV)
Fe
Cu
Ni
Fe
Fe
Ni
Cu
Ni
Cu
200 nm
(l)
Counts
Energy (keV)
Fe
Cu
Ni
Fe
Fe
Ni
Cu
Ni
Cu
200 nm

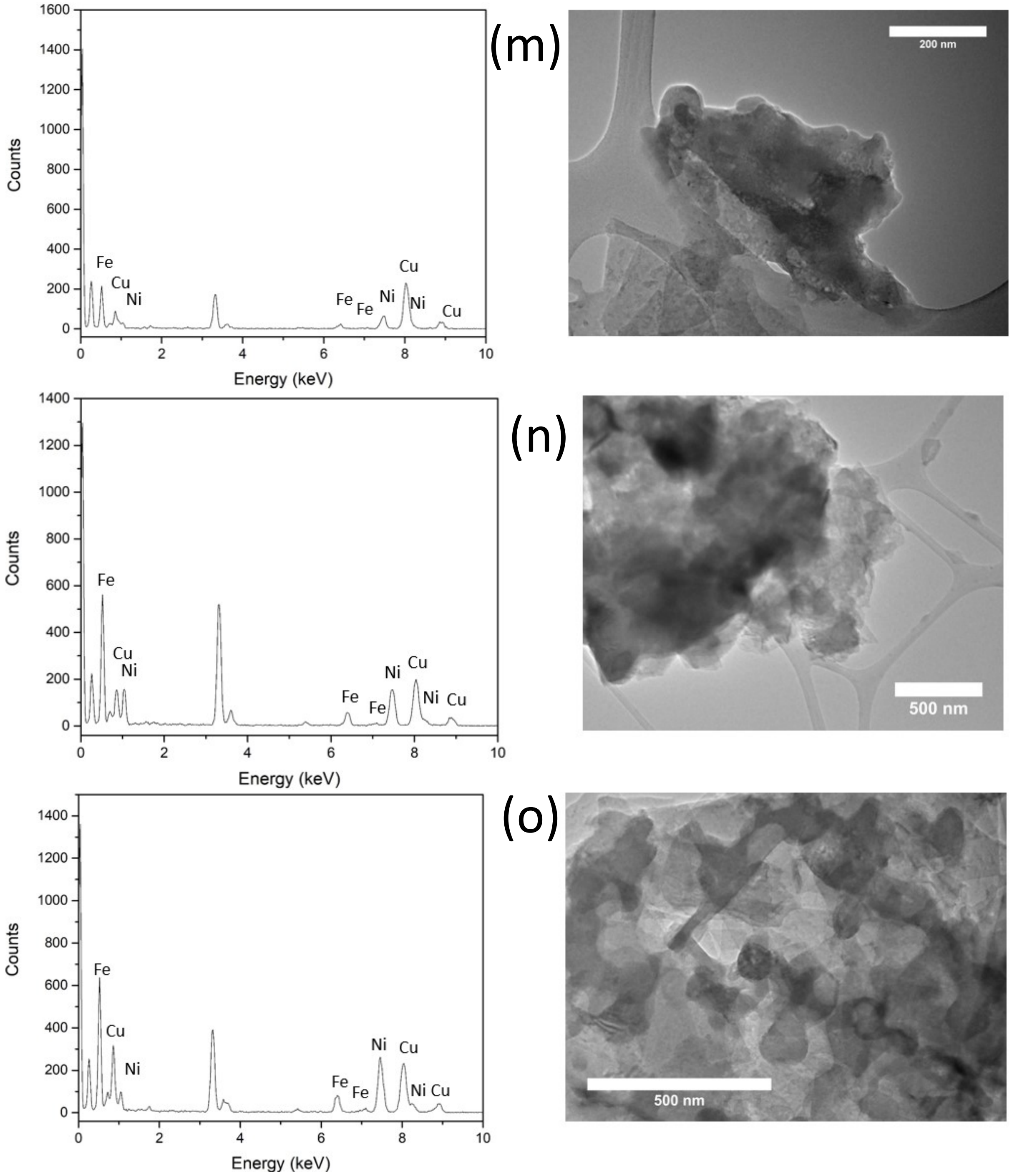

Figure 6: EDX spectra and accompanying STEM images for the post-cycled NiFe LDH.

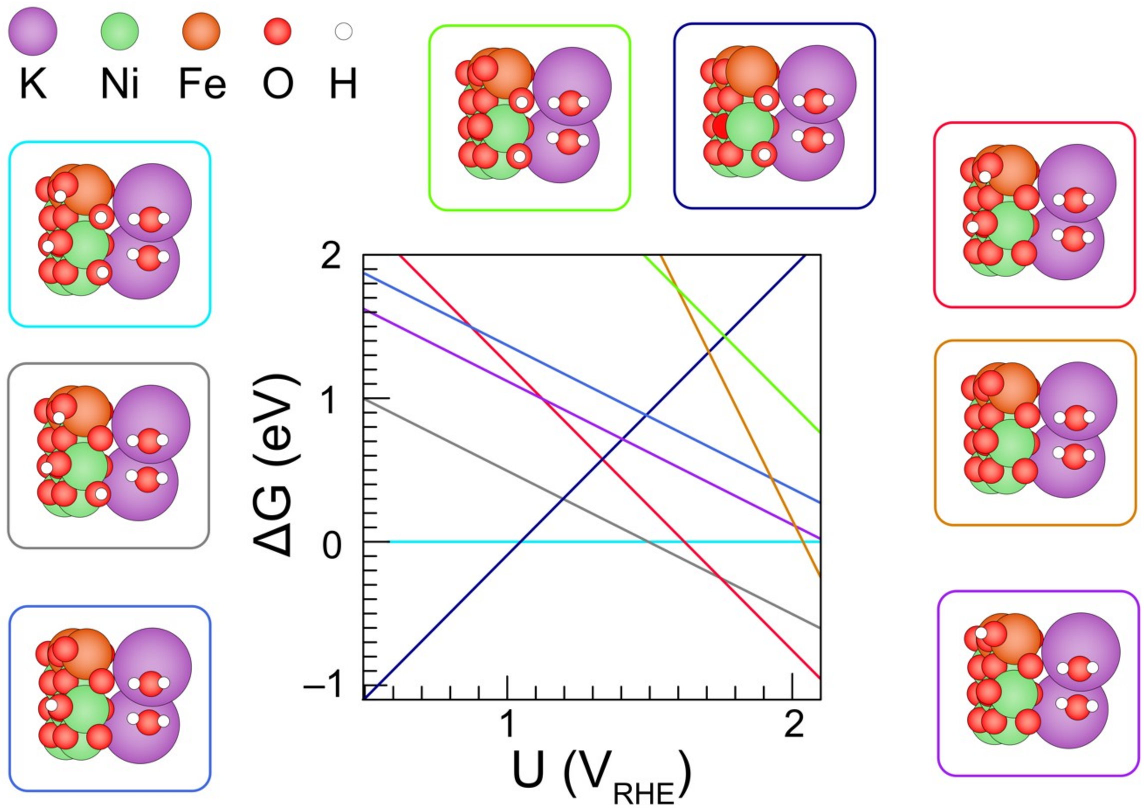

Figure 7: Considered coverages including those which are not the most stable, with the same legend as in Figure 7a in the main text.

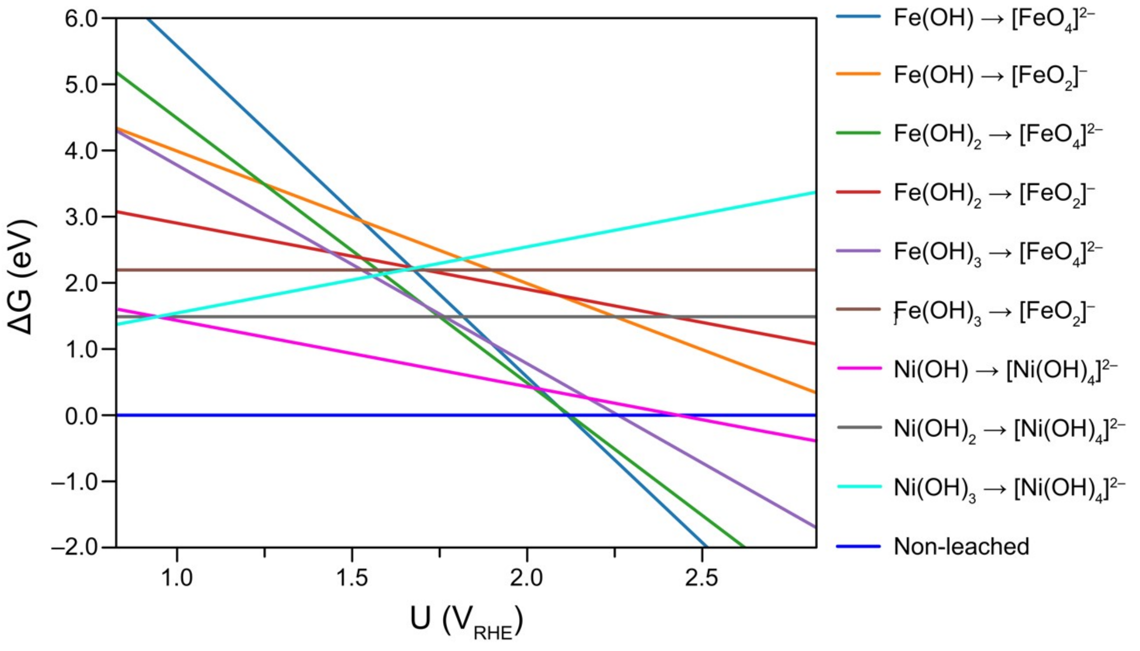

Figure 8: Leaching analysis for the $\theta_{2OH,2H}$ coverage where each line corresponds to the Gibbs formation energy of a given leached substance to the formation for any ionic species which is stable at pH = 14 from U=1-2 V_{SHE}.

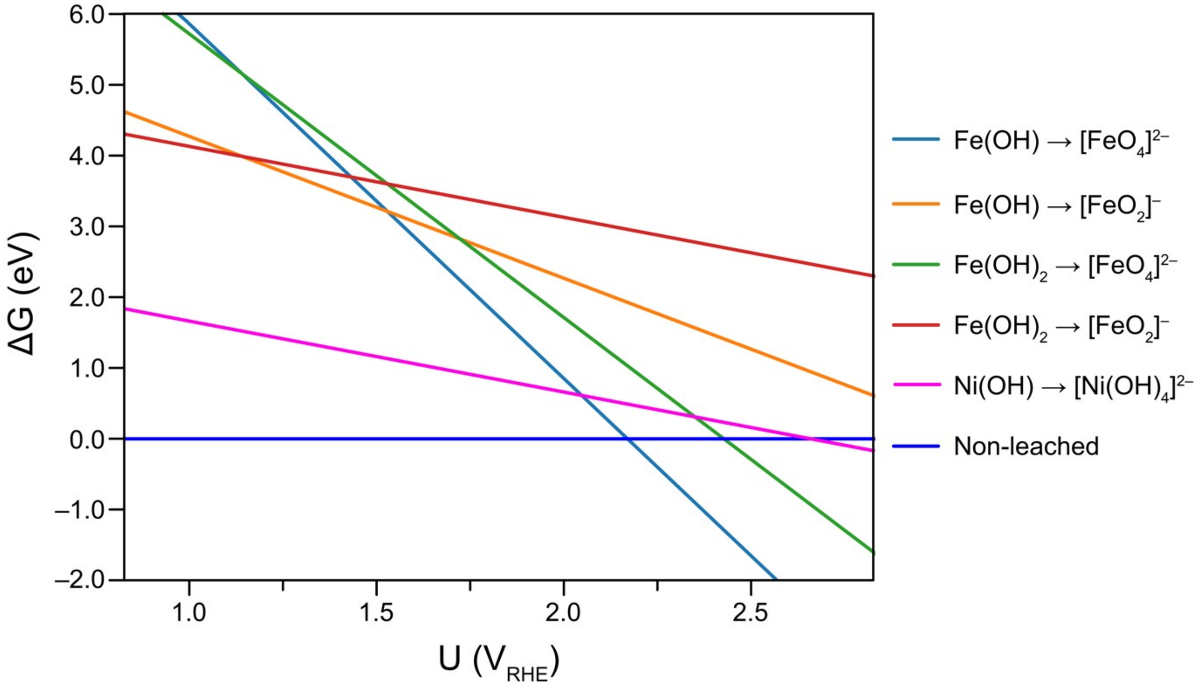

Figure 9: Leaching analysis for the $\theta_{2OH,1H}$ coverage where each line corresponds to the formation energy of a given leached substance to the formation for any ionic species which are stable from U=1-2 V, pH=14.

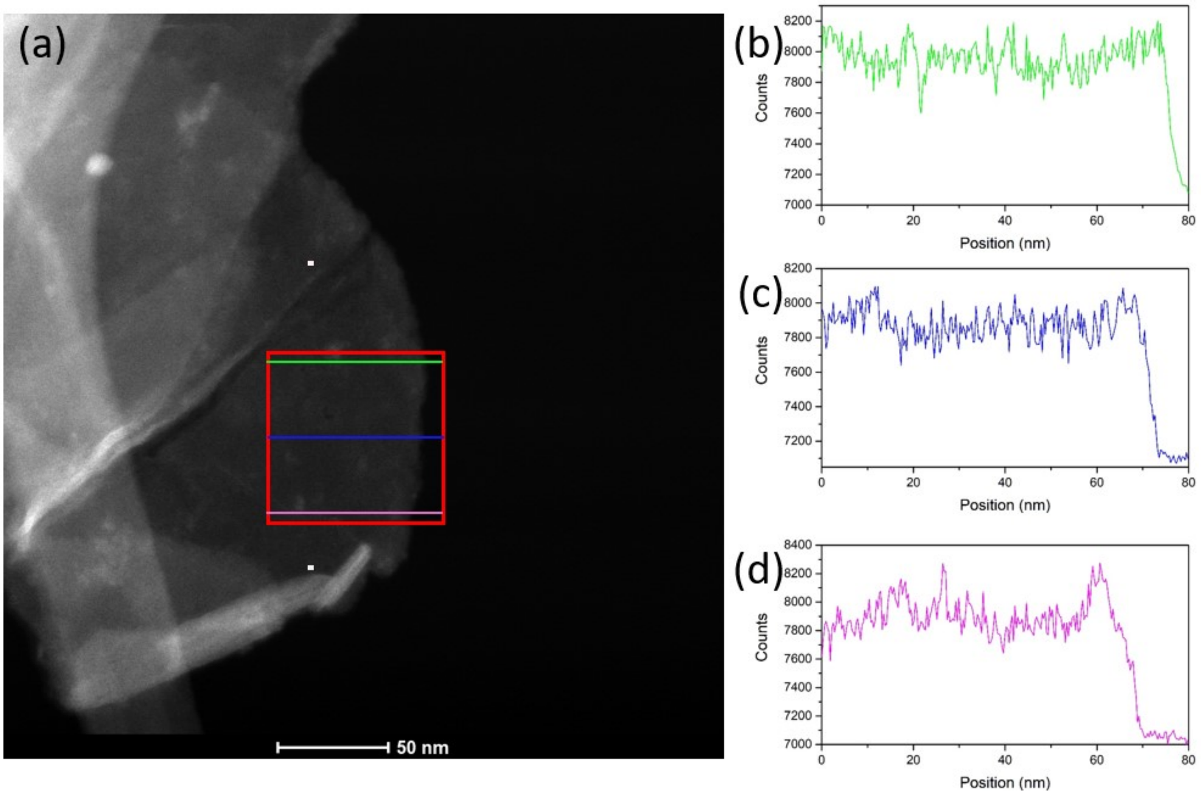

Figure 10: (a) STEM image showing the area of interest for EELS signal line scans (red) performed along the directions marked as (b) green, (c) blue and (d) pink. The spectra indicate relative uniformity of the signal, and hence thickness of the flake in the x-direction.

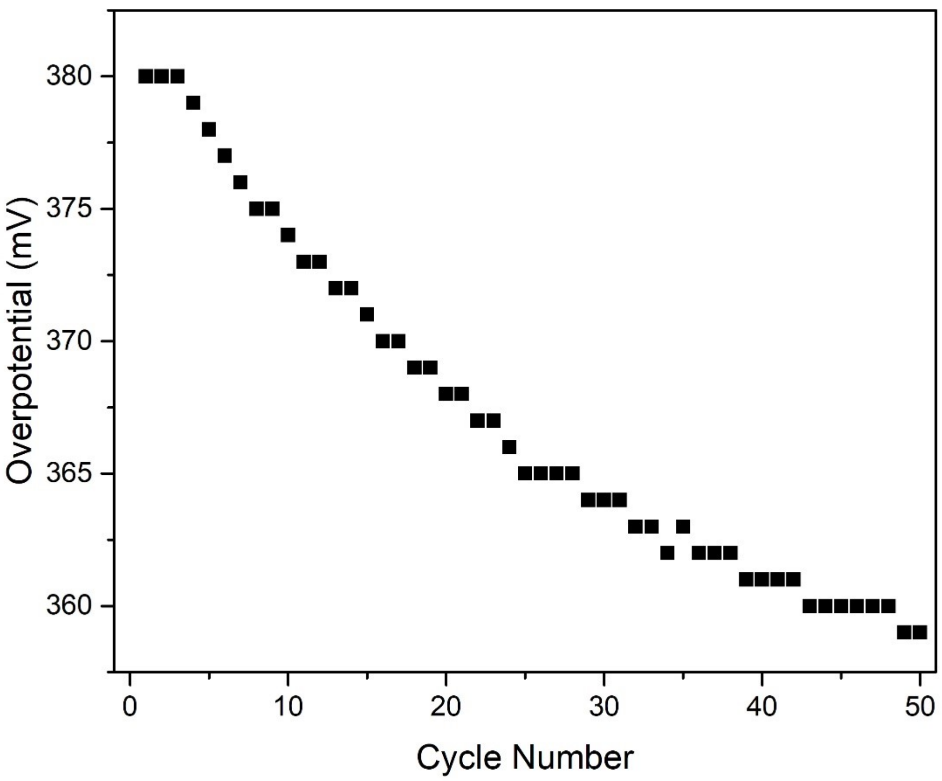

Figure 11: Overpotential (10 mA·cm^{-2}) values as a function of cycle number for a pure Ni foam catalyst in 1 M KOH, potential window 0 – 0.6 V vs Ag/AgCl.

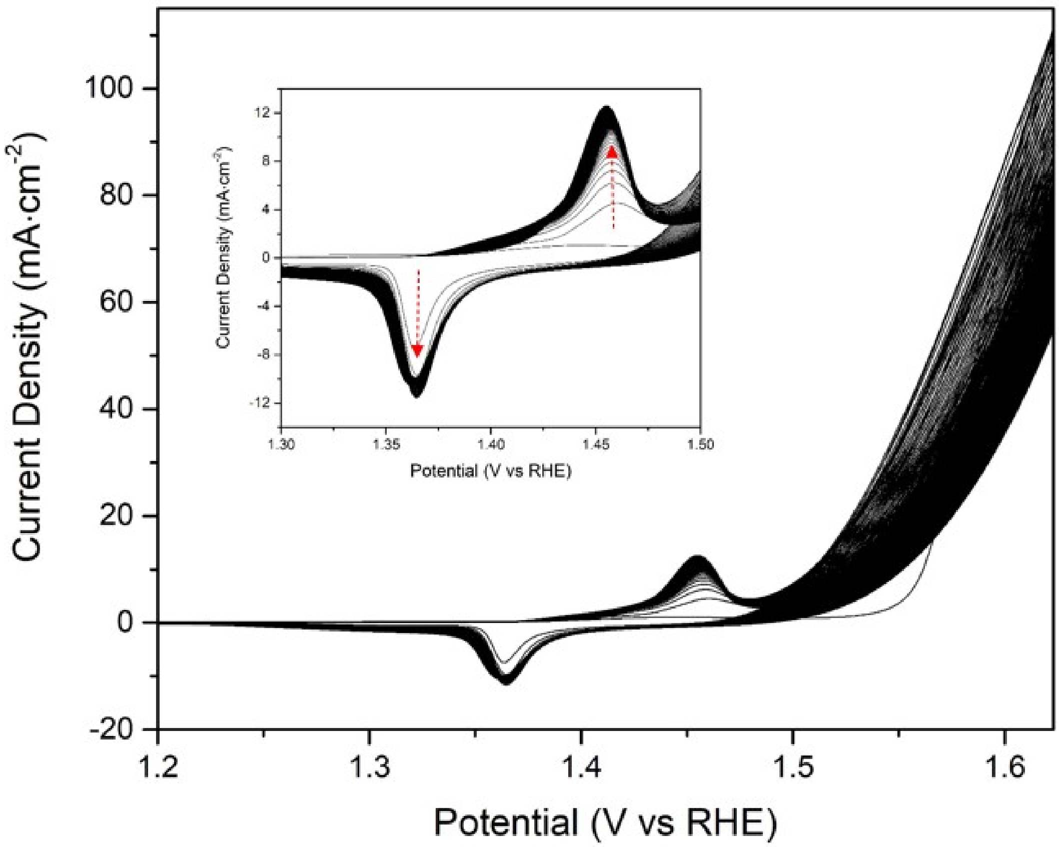

Figure 12: CV of a NiFe LDH-loaded Ni foam catalyst, cycled 200 times 0 – 0.6 V vs Ag/AgCl in 1 M KOH (inset: close-up view of the principle $Ni^{2+} \rightarrow Ni^{3+}$ redox couple emphasizing the gradual peak intensity growth).

Supplementary Tables

Table S1. *Zero-point energy (ZPE), heat capacity at constant volume (Cv), and entropic contributions (in eV) for adsorbates and gas molecules, calculated as outlined in the Computational Methods.*

**Adsorbate**	ZPE	C_V	$T\Delta S$
H_2O	0.57	0.10	0.67
H_2	0.27	0.09	0.57
HO*	0.37	0.04	0.06
O*	0.04	0.03	0.05
H*	0.30	0.00	0.01

Table S2: *ΔG values (in eV) calculated for Ni and Fe species at pH 14 from 0-2 V_{SHE}.*

**Element**	**Species**	ΔG°_{SHE}	ΔG_2
Ni	$[Ni(OH)_3]^-$	1.30[2]	$-2eU_{SHE} - 0.177pH + 0.026\ln a_{Ni(OH)_3^-} + 1.30$
	$[Ni(OH)_4]^{2-}$	2.13[2]	$-2eU_{SHE} - 0.236pH + 0.026\ln a_{Ni(OH)_4^{2-}} + 2.13$
Fe	$[FeO_2]^-$	1.10[3]	$-3eU_{SHE} - 0.236pH + 0.026\ln a_{FeO_2^-} + 1.10$
	$[FeO_4]^{2-}$	6.51[4]	$-6eU_{SHE} - 0.472pH + 0.026\ln a_{FeO_4^{2-}} + 6.51$
O	H_2O	0.00	$2eU_{SHE} + 0.118pH$
H	H_2O	0.00	$-eU_{SHE} - 0.059pH$

References

1. Rong, X.; Kolpak, A. M., Ab Initio Approach for Prediction of Oxide Surface Structure, Stoichiometry, and Electrocatalytic Activity in Aqueous Solution. *The Journal of Physical Chemistry Letters* **2015,** *6* (9), 1785-1789.
2. Bode, H.; Dehmelt, K.; Witte, J., Zur kenntnis der nickelhydroxidelektrode—I. Über das nickel (II)-hydroxidhydrat. *Electrochimica Acta* **1966,** *11* (8), 1079-1087.
